# Competition and interdependence define interactions of *Nostoc* sp. and *Agrobacterium* sp. under inorganic carbon limitation

**DOI:** 10.1038/s41522-025-00675-0

**Published:** 2025-03-08

**Authors:** Jonna E. Teikari, David A. Russo, Markus Heuser, Otto Baumann, Julie A. Z. Zedler, Anton Liaimer, Elke Dittmann

**Affiliations:** 1 Environmental Soil Science, Department of Agricultural Sciences, University of Helsinki, Helsinki, Finland; 2 Department of Microbiology, University of Helsinki, Helsinki, Finland; 3 Institute for Atmospheric and Earth System Research, University of Helsinki, Helsinki, Finland; 4 Bioorganic Analytics, Institute for Inorganic and Analytical Chemistry, Friedrich Schiller University Jena, Jena, Germany; 5 Department of Microbiology, University of Potsdam, Potsdam, Germany; 6 Department of Zoophysiology, University of Potsdam, Potsdam, Germany; 7 Synthetic Biology of Photosynthetic Organisms, Matthias Schleiden Institute for Genetics, Bioinformatics and Molecular Botany, Friedrich Schiller University Jena, Jena, Germany; 8 Department of Arctic and Marine Biology, University of Tromsø, Tromsø, Norway

**Keywords:** Soil microbiology, Biofilms

## Abstract

Cyanobacteria of the *Nostoc* genus are capable of forming symbiotic relationships with plants but also serve as a hub for heterotrophic bacteria. By comparing the axenic strain *Nostoc punctiforme* PCC 73102 and the xenic strains *Nostoc* sp. KVJ2 and KVJ3, we were able to demonstrate an almost obligate dependence of the cyanobacteria on the heterotrophic partners under carbon-limiting conditions. A detailed analysis of the intimate relationship between *N. punctiforme* and the isolate *Agrobacterium tumefaciens* Het4 using shotgun proteomics and microscopy uncovered a complex partnership characterized by competition for iron and facilitation for carbon. The prevalent extracarboxysomal localization of the carbon-fixing enzyme RubisCO suggests that a weak carbon-concentrating mechanism in *N. punctiforme* enforces a dependence on heterotrophic bacteria. Our study indicates a limited autonomy of symbiotic *Nostoc* strains, which may also explain its preference for symbiotic interactions.

## Introduction

Cyanobacteria are capable of maintaining a wide range of symbiotic relationships with eukaryotic hosts such as plants, fungi and protists^1^. Plant-associated *Nostoc* species play an essential role in terrestrial ecosystems, most importantly due to their ability to fix atmospheric nitrogen and further fuel the ecosystem by transferring newly assimilated nitrogen directly to their host plant^2^. In such epiphytic or endophytic symbioses, *Nostoc* releases most of the fixed nitrogen, mainly in the form of ammonia, to the host^3–5^. In return, the plant provides the cyanobacteria shelter and, more importantly, organic carbon^6^.

The ecology of the terrestrial and symbiotic *Nostoc* is generally well characterized. A complex lifestyle is maintained by large genomes that encode structures and physiological traits for specialized cells: nitrogen-fixing heterocysts; akinetes; and motile filaments, known as hormogonia, which have a great importance in the formation of the plant symbiosis^7,8^. *Nostoc* are also a rich source of bioactive natural products that have versatile but partially unknown roles in inter- and intraspecific communication within their ecosystem^9,10^.

Although cyanobacteria are photoautotrophic organisms, and thus generally considered independent of organic carbon sources, many of them are, in fact, facultative heterotrophs and dependent on the organic carbon supply from their ecosystem^11,12^. In symbiosis with plants, they take advantage of a heterotrophic lifestyle^13^ to allocate more energy for nitrogen fixation. Organic carbon is typically transported in small molecules such as glucose, but recent findings have shown that symbiotic *Nostoc* sp. have acquired a set of beneficial genes to transport and degrade more complex carbon sources, such as cell wall components^14,15^. The accumulation of beneficial genes over the course of evolution suggests plasticity in the carbon acquisition strategies, and the ability to utilize complex carbon sources may increase the fitness of cyanobacteria in plant symbiosis^15,16^.

In contrast, free-living *Nostoc* colonies, sometimes forming biofilms that may reach centimeters in diameter, rely on their own photosynthetic capacity and the availability of atmospheric CO_2_ which may become scarce in dense biofilms and large colonies. To cope better in low CO_2_ conditions, cyanobacteria have developed an effective carbon concentrating mechanism (CCM), that elevates CO_2_ near carboxysome-encapsulated RubisCO, the key enzyme involved in carbon fixation^17^. In marine and freshwater ecosystems, which are often C-limited in situ^18,19^, CO_2_ is also provided in exchange for dissolved organic carbon by the respiratory activities of heterotrophic bacteria tightly associated with cyanobacteria^20^. *Nostoc*-associated microbiomes have so far been studied for *Nostoc flagelliforme* and *Nostoc commune*, which form gelatinous colonies in soil, and for spherical *Nostoc* colonies in freshwater ecosystems^21,22^. While a pronounced habitat specificity was observed, the functional role of the bacteria in the *Nostoc* cyanosphere was not explicitly addressed. Therefore, the extent to which heterotrophic bacteria modulate the physiology of diazotrophic cyanobacteria remains largely unstudied. Notably, symbiotic *Nostoc* strains are difficult, sometimes impossible, to maintain as axenic isolates indicating intricate dependencies which are not yet understood^23^.

Here we specifically compared the influence of the heterotrophic microbiome on the growth of *Nostoc* at different inorganic carbon concentrations. We selected three symbiotic *Nostoc* species: axenic *Nostoc punctiforme* PCC 73102, and non-axenic *Nostoc* sp. KVJ2 and *Nostoc* sp. KVJ3^24^. Despite having a similar genetic repository for carbon acquisition, axenic cyanobacteria were unable to proliferate under inorganic carbon limitation, while non-axenic cyanobacteria thrived. Systematic investigation of the interaction between *N. punctiforme* PCC 73102 and the representative isolate *Agrobacterium tumefaciens* Het4 revealed a multifaceted relationship combining competition and facilitation for nutrients. The comparative analysis also provided insights into the weak carbon concentrating mechanism of *N. punctiforme*, which could foster the dependency relationships between the partners. Our study sheds new light on the limited autonomy of *Nostoc* strains and highlights the importance of the heterotrophic bacterial community for maintaining a vital ecosystem.

## Results

### Heterotrophic microbiome drastically enhances the vitality of *N. punctiforme* PCC 73102

**Fig. 1:**
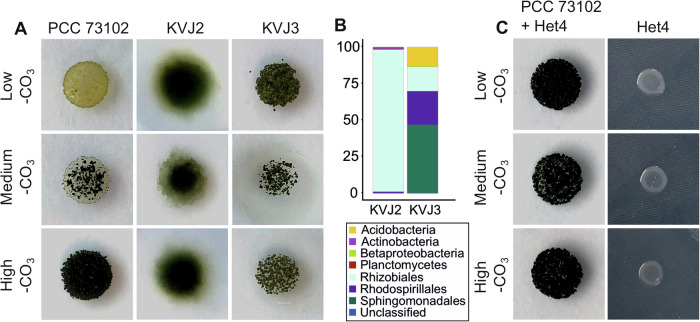
Growth of *Nostoc* sp. cyanobacteria is promoted by associated heterotrophic bacteria, particularly under inorganic carbon-limited conditions. **A** Axenic *N. punctiforme* PCC 73102 was unable to grow under low carbonate concentrations, whereas *Nostoc* sp. KVJ3 and KVJ2 thrived. Pictures were taken after seven days of cultivation on plates. **B** Microbiome analysis using 16S rRNA amplicon sequencing revealed a diversity of heterotrophic bacteria in *Nostoc* sp. KVJ2 and KVJ3, which were dominated by Alphaproteobacteria. **C** The isolate *A.tumefaciens* Het4, which was isolated from strain KVJ3 (Supplementary Fig. 1) promotes growth of *N. punctiforme* PCC 73102 under carbon-limited conditions. See Supplementary Fig. 3 for growth curves and digital image analysis.

Three *Nostoc* strains PCC 73102, KVJ2 and KVJ3 were grown on plates of BG11_0_ medium without a nitrogen source containing low, medium, or high carbonate concentrations. In case of low carbon conditions, Na_2_CO_3_ was omitted while medium concentrations correspond to the standard amount of Na_2_CO_3_ found in BG11_0_. For high carbonate conditions, Na_2_CO_3_ was added tenfold the amount of standard BG11_0_ medium. The growth was followed for eight days on three independent replicate plates. The axenic strain PCC 73102 showed virtually no growth without carbonate addition, slight growth under medium carbonate conditions, and rapid growth only at high carbonate concentrations (Fig. 1A). In contrast to axenic PCC 73102, strains KVJ2 and KVJ3, both carrying the associated microbiomes, grew surprisingly well even at low carbonate levels, and showed virtually no difference in growth between limiting and high carbonate concentrations. The dependence of PCC 73102 on very high carbonate concentrations indicated a weak CCM of the strain (Fig. 1A). Besides the differences in growth, the lack of motility of the axenic strain PCC 73102 compared to the strain KVJ2 was also noteworthy. Since the motility differences and the poor growth at lower carbonate conditions are less pronounced in the synonymous axenic strain ATCC 29133 maintained in the American Type Culture Collection^25,26^, phenotypic differences between PCC 73102 and the more recently isolated strains KVJ2 may also reflect different degrees of domestication.

To evaluate the adaptation strategies of the three studied *Nostoc* strains to niches with different inorganic carbon concentrations, the genetic repertoire of CCM-related genes was examined^27–29^. While the genome sequences of strain PCC 73102 and KVJ2 were publicly available in databases (GCA_000020025.1 and SAMN07173937), the sequence of strain KVJ3 was determined in the course of this study (PRJNA599284). All strains encoded the complete ATP-dependent bicarbonate uptake system BCT1 and BicA, along with NAD(P)H dehydrogenase complexes (Supplementary Table 1), which are important for the proper functioning of the bicarbonate uptake system^28^. However, the three strains did not encode for the highly conserved bicarbonate uptake transporter SbtA but only for a weakly homologous SbtA-like protein (Supplementary Fig. 1). This SbtA-like protein was recently annotated as putative SbtA for *N. punctiforme*^30^, but in fact shows only 24% identity to the authentic SbtA protein. Strains that encode the SbtA-like protein often carry the corresponding gene in addition to the canonical *sbtA* gene. This also applies to some *Nostoc* strains including the model strain *Nostoc* sp. PCC 7120, which, in addition to the SbtA-like protein (24% identity to SbtA of *Synechocystis* sp. PCC 6803), also encodes an SbtA orthologue (82% identity to SbtA of *Synechocystis* sp. PCC 6803, Supplementary Fig. 1). A physiological comparison of *Microcystis* strains that either carry both gene copies or only the *sbtA*-like gene indicated a weaker CCM for the latter, suggesting that the SbtA-like protein in *Microcystis* is not able to compensate for the absence of SbtA (Supplementary Fig. 1)^27^. We therefore assume that the absence of the canonical SbtA protein could be one of the reasons for the limited growth of the three *Nostoc* strains without additional inorganic carbon supply. Since all strains harbored the same set of genes encoding high- and low-affinity bicarbonate transporters, the vitality of the studied *Nostoc* cyanobacteria under inorganic carbon deficiency and their readiness to initiate plant symbiosis cannot be explained by a different genetic repertoire of CCM-related genes.

The genetic similarity in carbon acquisition and assimilation led us to anticipate that the heterotrophic bacterial communities play an important role in the vitality of free-living cyanobacteria under carbon limitation by providing CO_2_ and potentially other metabolites to the associated cyanobacteria. Thus, we studied the microbiomes of non-axenic strains KVJ2 and KVJ3 in more detail. Qualitative sequencing of 16S rRNA gene amplicons spanning the V3-V4 regions showed that both strains were heavily colonized by Proteobacteria (Fig. 1B, Supplementary Fig. 2). The microbiome of KVJ2 was more consistent, mainly comprising Rhizobiales, while the microbiome of KVJ3 had greater diversity, with additional presence of Sphingomonadales, Rhodospirillales and Acidobacteria.

Four different colony types of heterotrophic bacteria monocultures were isolated from KVJ3 (Het1-4) and three from KVJ2 (Het5-7) using R2A agar plates. Phylogenetic analysis of 16S rRNA gene sequences revealed that four isolates (Het1, 2, 4, 5 and 6) belonged to the *Rhizobium*/*Agrobacterium* group (Alphaproteobacteria), with Het1 and Het2 representing the same species (Supplementary Fig. 3). Thus, Het2 was omitted from further analysis. Het3 represented *Achromobacter* (Betaproteobacteria) genus whereas *Paenibacillus* sp. Het7, belonging to Firmicutes, was the only isolate beyond Proteobacteria. The isolation method used here showed clear selectivity towards *Rhizobium/Agrobacterium* and omitted Sphingomonadales and Acidobacteria, even though their abundance was evident based on the 16S rRNA amplicon metagenomic data. Notably, closely related strains of the *Rhizobium*/*Agrobacterium* group were also isolated after incidental laboratory contamination of the axenic strain PCC 73102 and isolated strains were included in the phylogenetic analysis as strains Het8 and Het9 (Supplementary Fig. 2).

Next, we tested the growth-promoting effects of the isolated heterotrophic bacteria on PCC 73102 by mixing axenic PCC 73102 with each of the isolates under BG11_0_ conditions. The growth was followed for eight days at medium carbonate concentration. Isolates Het1, 3, 4 and 8 strongly supported the growth of the cyanobacterium, and Het7 supported growth to some extent (Supplementary Fig. 3A). In co-cultures with Het5 and Het6, axenic cyanobacteria did not show signs of elevated growth, which points towards a great functional diversity of associated heterotrophic bacteria of the *Rhizobium*/*Agrobacterium* group.

The bacterial isolate *A. tumefaciens* Het4 (hereafter Het4) was selected to further understand the benefits of the microbial community for the cyanobacterium. This choice was based on the ability of Het4 to maintain physical interaction with PCC 73102 (Supplementary Fig. 3B) and to strongly enhance the growth of the cyanobacterium. The impact of the Het4 co-cultivation on growth of PCC 73102 was analyzed in replicate experiments for low, medium, and high carbonate concentration and quantified by digital image analysis. A pronounced growth promotion effect was particularly evident under medium carbonate conditions in BG11_0_ (Fig. 1C and Supplementary Fig. 4). In contrast, the opposite effect was found at high carbonate concentrations. While PCC 73102 grew faster under these conditions, the Het4 interaction had a negative effect on growth. These reciprocal growth effects indicate that Het4 can counteract C-limitation in particular, but that the interaction is not solely mutualistic (Supplementary Fig. 4, Supplementary Table 2). Next, we sequenced the genome of isolate Het4 (PRJNA599316). Analysis of amino acid identity (AAI) showed closest relatedness of the newly isolated strain to the *Agrobacterium* complex, which includes the well-known plant pathogen *Agrobacterium tumefaciens* str. C58 (Supplementary Fig. 5). Similar to strain C58^31^, the recently sequenced Het4 consisted of a circular chromosome (2.9 Mbp), a linear chromosome (2.1 Mbp), a cryptic plasmid (0.4 Mbp) and a tumor-inducing plasmid (0.37 Mbp). Het4 showed a very broad potential for the utilization of sugars, sugar alcohols and aromatic compounds as described for the reference strain C58. Further, Het4 carried the *nos* operon, which is required for the final step of the full denitrification pathway, and C58 is capable of directly ammonifying nitrate and nitrite. Neither Het4 nor C58 had known pathways for nitrogen or carbon fixation.

### Heterotrophic bacteria modulate the physiology of *N. punctiforme* PCC 73102

To gain a better understanding of the interaction between Het4 and the cyanobacterium, the PCC 73102 proteome was compared in the presence and absence of Het4 using a shotgun proteomic approach. To this end, samples for endoproteome analysis were collected after eight days of cultivation on BG11_0_ agar plates with and without Het4. In total, 4078 proteins were identified in the PCC 73102 monoculture and 4062 were identified in co-culture with Het4. 4023 proteins were identified in both conditions and, in the presence of Het4, 123 proteins were significantly upregulated and 117 proteins were downregulated (FC ≤ −2 & ≥2 and adjusted *p*-value < 0.05) (Supplementary Fig. 6 and Supplementary Dataset 1). Remarkably, amongst the set of upregulated proteins, 17 phycobilisome subunits were identified (Supplementary Fig. 6 and Supplementary Dataset 1). This supports the differences observed in the greenness of mono- and co-culture where PCC 73102 exhibits strong bleaching when grown in low/medium inorganic carbon concentrations in the absence of Het4 (Fig. 1A, C).

**Fig. 2:**
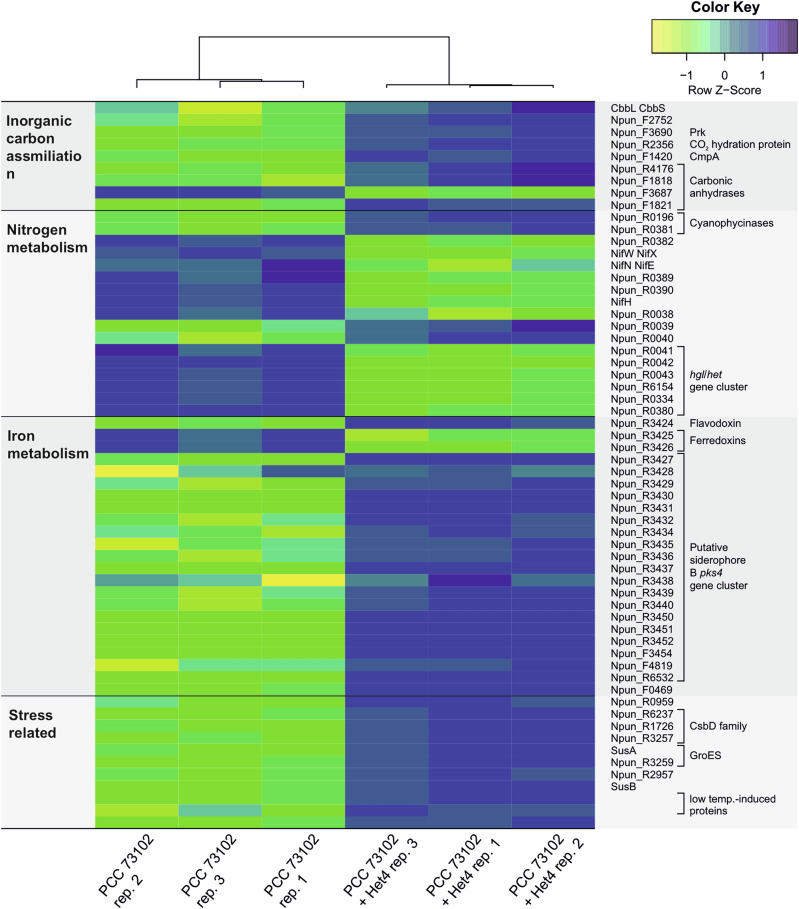
Heatmap illustrating the differential expression of selected proteins between *Nostoc punctiforme* PCC 73102 monoculture and co-culture with *A. tumefaciens* Het4. Rows represent individual proteins, while columns represent biological replicates of each condition. Proteins are labeled with gene names (right) and grouped by predicted cellular function (left). Log2 protein expression levels were transformed with a z-score normalization and represented with a color scale that varies from yellow (most downregulated) to blue (most upregulated) relative to the mean expression across all samples in each row. Hierarchical clustering was performed on samples and the dendrograms indicate the similarity between them.

Given the ability of Het4 to complement the weak CCM of PCC 73102, we were anticipating an N for C exchange between the diazotrophic cyanobacterium and Het4. Therefore, we first investigated protein categories representing carbon fixation, the carbon concentrating mechanism and nitrogen fixation. While the large and small subunit of RubisCO and phosphoribulokinase (Npun_F2752) were only slightly upregulated in the co-culture, we found a pronounced upregulation of three carbonic anhydrases (CAs) (Npun_F1420, Npun_R4176, Npun_F3687), one bicarbonate transporter (Npun_R2356), and one CO_2_ uptake transporter (Npun_F3690) (Fig. 2). This observation supports the hypothesis that Het4 indeed provides CO_2_ to the cyanobacterium, especially because the strongest upregulation was observed for the predicted periplasmic CA (Npun_F3687) (Fig. 2). In contrast, proteins involved in N-fixation behaved somewhat contrary to our assumptions. While the nitrogenase NifH itself was slightly upregulated in co-culture, accessory proteins such as the iron-molybdenum cofactor biosynthesis proteins NifN and NifE, and the stabilizing protein NifW, were clearly downregulated. Even more surprising was the strongly reduced expression of heterocyst glycolipid biosynthesis proteins (Npun_R0038-R0043) pointing to a downregulation of heterocyst formation and N-fixation. The unexpected nature of these findings raises some questions on the nitrogen source under the given diazotrophic conditions. Notably, we observed an upregulation of two cyanophycinases (Npun_F1821 and Npun_R0196). Cyanophycinases break down the multi-L-arginyl-poly-L-aspartic acid (cyanophycin) reserve material of cyanobacteria^32^ suggesting that the potential impairment of nitrogen fixation is at least partially compensated by the utilization of nitrogen storage compounds (Fig. 2).

In the search for possible reasons for the partial downregulation of N-fixation, we were particularly struck by the upregulation of flavodoxin (Npun_R6154), which was the top hit among the upregulated proteins and is a marker for iron limitation^33^ (Supplementary Fig. 6). Flavodoxins function analogously to ferredoxins as electron transfer proteins, but unlike ferredoxins are not dependent on iron. The parallel downregulation of two of the ferredoxins (Npun_R0334 and Npun_R0380) suggests a functional replacement of ferredoxins by flavodoxin in co-culture due to iron deficiency (Fig. 2). This hypothesis was further fueled by the observed upregulation of the cryptic siderophore cluster PKS4 (Npun_R3414-Npun_R3453)^34^ and its TonB-dependent siderophore receptor (Npun_R3454) (Fig. 2B). These data point to a competition for iron between PCC 73102 and Het4. As adverse effects of iron-limitation on nitrogen fixation are well known^35^, this competition may account for the preferential use of stored nitrogen over nitrogen fixation. The comparative proteomic study also revealed further evidence of negative interactions between the strains. For example, a number of stress markers were over-accumulating in the co-culture, in particular proteins of the cold shock protein family CsbD (Npun_R6532, Npun_F0469 and Npun_R0959), but also markers for osmotic stress such as a sucrose synthase (SusA and SusB) and chaperones of the GroES family (Npun_R6237 and Npun_R1726) (Fig. 2). These findings only make it more astonishing that, overall, the dominant interaction is the mutualistic growth promotion of PCC 73102. Under standard BG11_0_ growth conditions, and without heterotrophic support, PCC 73102 shows severe signs of starvation evident by phycobiliprotein degradation. However, if the C-limitation is compensated by high carbonate, the negative effects of the co-cultivation dominate (Supplementary Fig. 4).

Since the better growth of the PCC 73102-Het4 co-culture on agar plates could also be due to stimulated biofilm formation, in which Het4 might compensate for the pronounced domestication of PCC 73102, we also compared marker proteins for hormogonia, chemotaxis, and pili. We found several taxis-related proteins being upregulated in the presence of Het4, specifically proteins encoded by the Hmp locus (Npun_F5960- F5964), and the alternative chemotaxis locus Npun_F5636-F5640, but also the PtxB protein (Npun_F2162) and the CheB-like protein Npun_R 0244 (Supplementary Fig. 7)^25^. Notably, proteins of the nostopeptolide locus (Npun_F2181-Npun_F2186) were also upregulated. The product of this gene cluster stimulates or inhibits hormogonium formation in a concentration-dependent manner^10^. In contrast, the influence of Het4 on pili proteins was less pronounced, with only a weakly negative influence on the main PilA1 protein (Npun_F0676) and the extension protein PilB (NpunR_0118) detectable. Only pili proteins PilM and PilQ (NpunF_5005 and NpunF_5008) involved in early stages of pili assembly were significantly upregulated in response to the presence of Het4 (Supplementary Fig. 7)^26^. Taken together, Het4 stimulates the upregulation of chemotaxis-related proteins and part of the pili apparatus, thereby possibly also promoting a better growth of PCC 73102 on agar plates.

### Phenotypic response to inorganic carbon limitation in *N. punctiforme* PCC 73102 in mono- and co-cultures

Given that the strong dependence of the cyanobacterium on heterotrophs can primarily be explained by a weak CCM, we initiated phenotypic studies on the subcellular localization of RubisCO using immunofluorescence microscopy (IFM) and transmission electron microscopy (TEM).

**Fig. 3:**
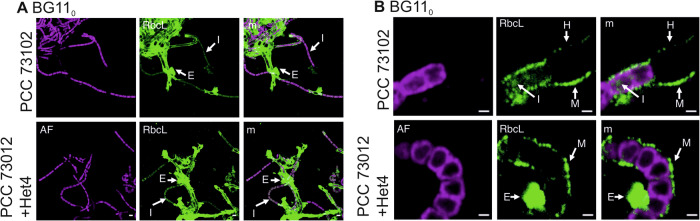
Immunofluorescence microscopy (IFM) images showing subcellular localization of RbcL, the large subunit of RubisCO, in *N. punctiforme* PCC 73012 monocultures and co-cultures with *A. tumefaciens* Het4 under nitrogen-deplete (BG11_0_) conditions. **A** shows an overview of micrographs illustrating the phenotypic heterogeneity among filaments either with predominant extracellular (E) or intracellular localization (I) of RubisCO. **B** depicts selected detail images showing subcellular localization of RbcL in mono- and co-cultures. Three localization types, extracellular, near membrane, and cytosolic RbcL were found in both cultures with and without heterotrophic bacterium; however, extracellular RbcL was more abundant in co-cultures, especially under nitrogen-deplete conditions. M membrane-near RbcL, I intracellular RbcL, E extracellular RbcL H heterocyst, AF autofluorescence, m merged. Scale bar **A**: 5 µm; scale bar **B**: 1 µm.

IFM with an antibody against the RubisCO subunit RbcL revealed a pronounced phenotypic variability in the subcellular localization of RbcL at all studied conditions. In addition to intracellular localization, we observed many filaments with a pronounced extracellular localization of RbcL (Fig. 3A). Furthermore, the intracellular signals often occurred at the cell periphery (Fig. 3B). Overall, we found little evidence for carboxysomal localization of RubisCO. To exclude methodological bias, we also labeled the carboxysomes with a CcmK antibody which detects the shell protein of the cyanobacterial microcompartments^36^ and were able to detect the typical carboxysomal structures (Supplementary Fig. 8). Even though RubisCO may be less accessible to the antibody in the carboxysomes, it can be clearly stated that large amounts of RubisCO are localized outside the carboxysome in PCC 73102 under the conditions tested. Control experiments with an anti-GFP antibody showed no signals at any of the above sites (Supplementary Fig. 9), confirming the specificity of the RbcL signal. In axenic cultures, RbcL was found mainly in the cytoplasm of the cells as well as near the cell membrane, but extracellular RbcL was also identified to some extent (Fig. 3A, B, upper panel). In co-cultures with Het4, RbcL was more commonly detected in the extracellular sheath and, at a lower amount, intracellularly (Fig. 3A, B, lower panel). Interestingly, phenotypic plasticity of RbcL localization was also seen among filaments located next to each other, indicating strong heterogeneity within the culture under nitrogen deficiency (Fig. 3A).

To confirm the extracellular accumulation of RubisCO, we performed a second proteomic study in which we characterized the extracellular proteome of PCC 73102 using the recently described EXCRETE method^37^. In total, 2970 proteins were identified in the PCC 73102 monoculture and 3085 were identified in co-culture with Het4. 2896 proteins were identified in both conditions. Overall, the large number of proteins identified in the exoproteome under BG11_0_ conditions, and the major overlap with the endoproteome, indicated a possible lysis of part of the PCC 73102 cells (Supplementary Fig. 10 and Supplementary Dataset 2). Specifically, RubisCO was detected among the ten most abundant proteins in the medium, both in mono- and co-culture with Het4 along with further abundant intracellular proteins such as phycobilisome subunits and the periplasmic carbonic anhydrase (Supplementary Fig. 10). The strong extracellular accumulation of RubisCO is apparently specific for N- and C-limiting conditions, since a recently published exoproteomic study of PCC 73102 showed that under N- and C-replete conditions predicted extracellular proteins were enriched, but not RubisCO^37^. To evaluate whether the exoproteomic difference between nitrogen-deplete (BG11_0_) and nitrogen-replete (BG11) conditions is also reflected in the extracellular localization of RubisCO detected by IFM, we also transferred mono- and co-cultures from BG11_0_ conditions to BG11 conditions and evaluated them by IFM after eleven days (Supplementary Fig. 11). Indeed, the apparent proportion of external RbcL decreased, while the intracellular RbcL content in vegetative cells increased thereby supporting the findings of the exoproteomic analyses (Supplementary Fig. 11 and ref. ^37^). This phenomenon suggests that RubisCO is primarily accumulating outside the cells under inorganic carbon and nitrogen deficiency.

Notably, RubisCO was not differentially accumulating in the exoproteome of the mono- and co-culture with Het4 (Supplementary Fig. 10 and Supplementary Dataset 2). We assume that part of the extracellular RubisCO which sticks to the cell-bound mucus layer, especially in the co-culture (Fig. 3A), is not enriched by the exoproteomic method used here but remains in the cellular pool. Nonetheless, the characterization of the exoproteome provides further evidence that *N. punctiforme* suffers from starvation under standard BG11_0_ growth conditions and may sacrifice cells and proteins including RubisCO to increase the viability of the community.

**Fig. 4:**
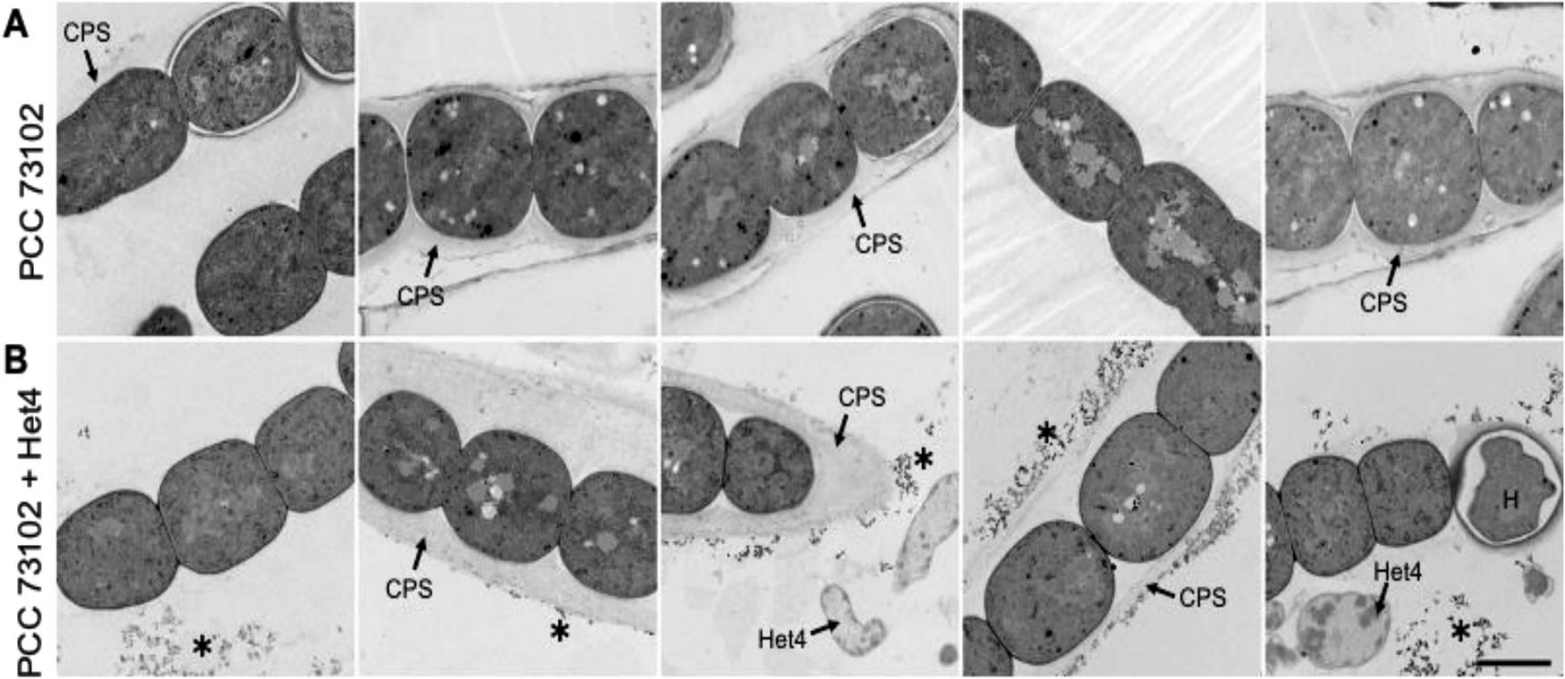
Transmission electron microscopy (TEM) images of *N. punctiforme* PCC 73102 grown in monoculture or co-culture with *A. tumefaciens* Het4 under nitrogen-deplete conditions. **A** TEM images of axenic *N. punctiforme* PCC 73102 show heterogeneity in the polysaccharide capsule. **B** TEM images of *N. punctiforme*-Het4 co-cultures show heterogeneity in the polysaccharide capsule and an accumulation of granular material (highlighted by an asterisk) predominantly in the periphery of the polysaccharide layer. CPS capsular polysaccharides, H heterocyst. Scale bar = 2 µm.

**Fig. 5:**
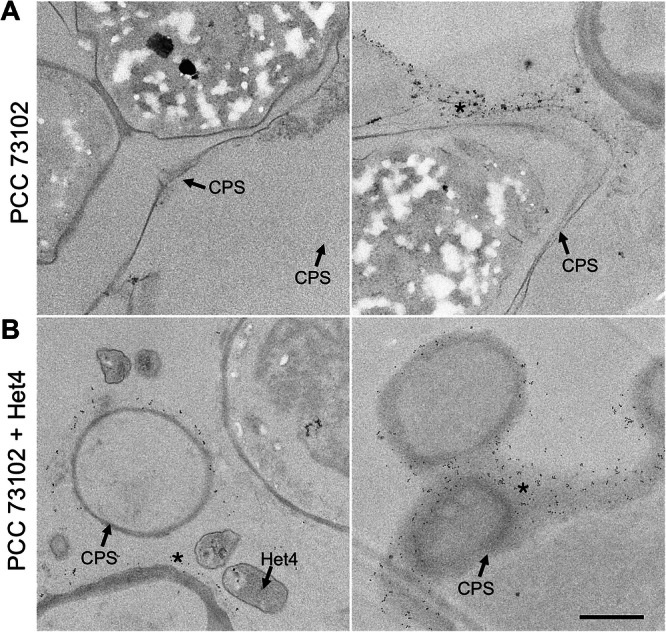
Immunogold TEM images of *N. punctiforme* PCC 73102 monoculture and PCC 73102-Het4 co-culture samples incubated with antibody against RubisCO subunit RbcL. **A** *N. punctiforme* PCC 73102 monoculture and **B** PCC 73102-Het4 co-culture samples. Immunogold particles are visible at the periphery of the polysaccharide capsule (CPS) in both mono- and co-culture (indicated by an asterisk), but they are much more abundant in the co-culture with Het4. Scale bar: 1 µm.

To gain further insight into ultrastructural differences within cyanobacterial cultures and between axenic and co-cultures under inorganic carbon limitation, we used TEM. Interestingly, there was structural heterogeneity with respect to the capsular sheath (CPS) among PCC 73102 filaments within and between axenic and co-cultures (Fig. 4). The presence of the capsular sheath varied, with some filaments showing no detectable sheath, while others had a relatively electron-lucent or compacted sheath that was up to 2 µm thick. We had the impression that the capsular sheath of PCC 73102 was more prominent in co-cultures. In addition, we observed small electron-dense granular particles outside the PCC 73102 cells, often associated with the outer surface of the capsular sheath, and these particles were present at a far larger amount in co-cultures (Fig. 4B). Sheathed cells contained an increased number of small electron-dense granular particles outside the cells. The presence of external RbcL in the granular particles was confirmed by pre-embedding immunogold TEM (Fig. 5). In co-cultures, large amounts of extracellular RbcL could be detected in the periphery of the cyanobacterial sheath, similar to the IFM studies under the same conditions (Figs. 3 and 5). Control experiments with an anti-GFP antibody showed no signals at any of the above sites (Supplementary Fig. 12).

## Discussion

The present study not only provides detailed insights into the intimate relationship of the symbiotic strain *N. punctiforme* PCC 73102 and its natural heterotrophic partner *Agrobacterium tumefaciens* Het4, but also demonstrates the limited autonomy of the diazotrophic photoautotrophic cyanobacterium under N- and C-deficient conditions. We showed that a combined C and N limitation enforces an almost obligate dependence of PCC 73102 on heterotrophic partners. It is particularly remarkable that C-limitation manifests itself already at the commonly used inorganic carbon concentrations of the BG11_0_ standard medium. Under these conditions, the axenic strain PCC 73102 shows clear signs of bleaching and a pronounced accumulation of intracellular proteins in the exoproteome, that likely results from starvation and cell lysis. However, the carbonate-dependent growth deficiencies are contrasting experiences with the cultivation of the synonymous strain ATCC 29133, which not only shows pronounced motility on agar plates but also grows in media without added carbonate, such as Allen and Arnon medium^25^. These phenotypic deviations suggest significant differences in the domestication of the two strains, not only with regards to motility, which is often impaired in laboratory cultures^38^, but possibly also in relation to the CCM. A stronger dependence on high inorganic carbon concentrations is presumably promoted by decades of cultivation with carbonate and bicarbonate at the Pasteur Culture Collection. Yet, we assume that the growth reduction caused by the absence of SbtA occurs independently of the differences in domestication. Both the PCC 73102 strain, which depends on the addition of carbonate, and the KVJ2 strain, which also grows on plates without the addition of carbonate, grow much better with high CO_2_ supply, as we have demonstrated recently in a study using high-density cultivation developed by the CellDeg GmbH^39^. However, the almost obligate dependence on heterotrophic bacteria observed by us primarily applies to the strain PCC 73102 and the selected cultivation conditions in this study. This conclusion is also supported by the fact that a considerable number of *Nostoc* strains could be axenically isolated^40,41^.

**Fig. 6:**
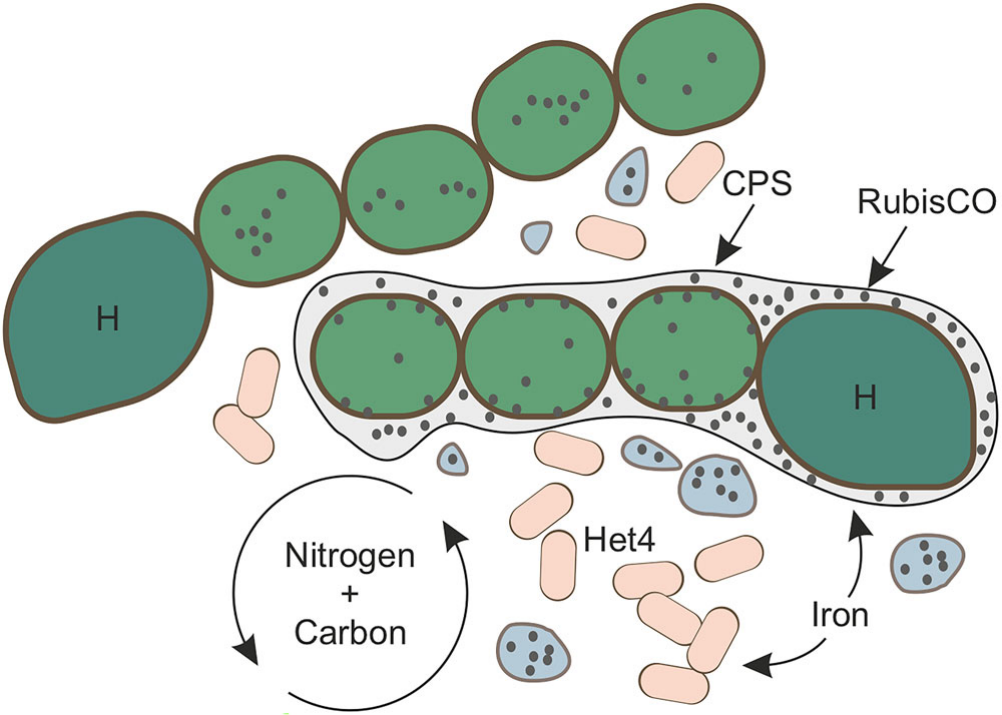
Model for the interaction between *N. punctiforme* PCC 73102 and *A. tumefaciens* Het4 under N- and C-limiting conditions.

The fact that the dependence of PCC 73102 on heterotrophic bacteria can be abolished by addition of high carbonate concentrations (Fig. 1A) indicates a weak CCM in *N. punctiforme* compared to model cyanobacteria such as *Synechocystis* sp. PCC 6803. In the search for causes, we discovered differences in the genetic repertoire of bicarbonate uptake transporters, in particular a lack of the gene encoding the high-affinity uptake transporter SbtA. Although the three *Nostoc* strains encode for a weakly homologous SbtA-like protein, their ability to concentrate bicarbonate is likely lower compared to strains encoding both SbtA and the SbtA-like protein such as the widely studied strain *Nostoc* sp. PCC 7120. Furthermore, we detected a predominantly extracarboxysomal localization of RubisCO, often in the cytoplasmic membrane region. Both findings are similar to what is known for the bloom-forming freshwater cyanobacterium *Microcystis aeruginosa* PCC 7806^42^. Notably, the Pasteur Culture Collection recommends the addition of NaHCO_3_ to standard BG11/BG11_0_ medium for both *Microcystis* and *N. punctiforme* for axenic maintenance (https://catalogue-crbip.pasteur.fr/). It is tempting to speculate that these cyanobacteria are more specialized than unicellular model cyanobacteria on utilizing respiratory CO_2_ from heterotrophic bacteria and are therefore less dependent on a strong CCM in nature. The cytoplasmic membrane localization of RubisCO may facilitate the intimate inorganic carbon supply. This hypothesis is supported by the proteomic findings in the present study. In particular, the activation of carbonic anhydrases and bicarbonate or CO_2_ transporters in co-cultures with Het4 is striking. The benefits of this inorganic carbon feeding by Het4 are clearly evident through growth promotion, even against the background of negative interactions, including competition for iron (Fig. 6).

In addition to its dependence on inorganic carbon supply by heterotrophic partners, a pronounced phenotypic plasticity of the PCC 73102 filaments is also noteworthy in the present study. This plasticity was observed with regard to the extent and nature of the extracellular sheath as well as with regard to the subcellular localization of RubisCO and the accumulation of RubisCO-containing extracellular granules. As we did not synchronize our cultures for the experiments presented in this study, the phenotypic plasticity may also reflect asynchronous cycling given the complexity of the organism and its developmental program^7^. The extracellular sheath of microalgae is commonly referred to as the phycosphere and provides a nutrient-rich microscale interface favoring phototroph-heterotroph interactions^43^. The plasticity may reflect a division of the population into filaments specializing on cooperation with Het4 and those that thrive independently of it. Division of labor and cheating on resources are common phenomena in mono- and multispecies biofilms and may also contribute to versatility and resilience of the *Nostoc* population^44,45^. As we have no global proteomic insight into the differences between the distinct filament types, we can only speculate about traits favoring cooperation. The increased accumulation of RubisCO-containing extracellular granules around sheathed filaments in the co-culture may indicate a major role of protein recycling for the cooperation between PCC 73102 and Het4. RubisCO recycling has been shown to play a major role in plants where nutrient starvation leads to the release of RubisCO-containing particles (RCBs) from chloroplasts as part of an autophagic process^46^. The denitrification potential of Het4 may contribute to the N recycling process. Notably, an enrichment of denitrifying heterotrophic bacteria was also observed in diazotrophic freshwater cyanobacteria of the genus *Dolichospermum* under N-limitation^47^.

The extracellular mucus layer may also be one of the reasons for the specificity of *Nostoc* interactions with its heterotrophic microbiome. Even though we have studied only two field strains, the observed major differences in their heterotrophic communities support a host specificity in principle. Previous studies have suggested that Rhizobiales generally play important roles in plant-cyanobacterium consortia, and our data strongly support these findings^48^. Yet, the impact of *Rhizobium*/*Agrobacterium* on PCC 73102 was clearly strain-specific as the closely related Het6 isolate did not show signs of growth promotion (Supplementary Fig. 3). The bilateral selectivity of the growth promotion indicates that variable functional traits, that are not part of the species’ core genome, may be crucial for the interaction.

The present study sheds new light on the dependence of symbiotic cyanobacteria of the genus *Nostoc* on respiratory activities of associated heterotrophic bacteria. This dependence is likely enforced by a weak CCM and is promoted by a physical interaction around the pronounced sheath of the cyanobacteria. Symbiotic *Nostoc* strains apparently rely on partners for the supply of carbon, either heterotrophic bacteria in free-living *Nostoc* colonies that provide CO_2_ or plant hosts that provide organic carbon. This view is supported by a recent study showing that *Nostoc* strains in cycads grow largely without heterotrophic bacteria, whereas after isolation from their plant partner they form consortia with heterotrophic bacteria^49^. Although the mutualistic interactions of *Nostoc* and *Agrobacterium* have been observed mainly under N and C deficiency, it can be assumed that they play a major role in many habitats and strongly influence the vitality of *Nostoc* colonies. Understanding the interdependencies is not only important for assessing the role of phototroph-heterotroph interactions in nutrient cycles, but also central for the biotechnological exploitation of *Nostoc* strains.

## Methods

### Strains and growth conditions

Axenic *Nostoc punctiforme* PCC 73102 (hereafter PCC 73102) was obtained from the Pasteur Culture Collection and maintained in liquid BG11_0_ medium^50^ under continuous illumination of 25 µmol photons m^−^^2^s^−^^1^ at 23 °C. *Nostoc* sp. KVJ2 (hereafter KVJ2) and *Nostoc* sp. KVJ3 (hereafter KVJ3) were isolated from Northern Norway as described previously^24^ and since then cultivated diazotrophically in BG11_0_ medium under continuous light of 25 µmol photons m^−^^2^s^−^^1^ at 23 °C. Heterotrophic bacteria (Het1-6) were isolated from the strains KVJ2 and KVJ3 by plating the culture on R2A agar plates and purified over several cultivation cycles at 23 °C.

### Plate assay

Effects of inorganic carbon concentrations on the growth of cyanobacteria on agar plates were studied in three different carbonate concentrations: low (BG11_0_ without added Na_2_CO_3_), medium (BG11_0_ containing 0.19 mM Na_2_CO_3_) and high (BG11_0_ enriched with 1.9 mM Na_2_CO_3_). Concentration of the sodium ions in low bicarbonate medium was adjusted by NaCl. Prior to the experiment, PCC 73102, KVJ2 and KVJ3 cyanobacteria were washed twice using low carbonate BG11_0_ medium, resuspended in the same medium (OD750 = 4.4) and a volume of 10 µL each was dropped onto three agar plates in three biological replicates. To study the growth promotion of heterotrophic bacteria, heterotrophic bacteria were washed and resuspended in a similar concentration as cyanobacteria (final OD600 = 2.2) and 10 µL of the bacteria were added on the top of the cyanobacterial drop (physical interaction) and cultivates at 23 °C in continuous illumination of 15–23 µmol photons m^−^^2^s^−^^1^ for seven days.

### Cultivation in liquid cultures

For the immunofluorescence and electron microscopy, and proteomic analysis liquid culture experiment was carried out. Prior to the experiments, PCC 73102 was washed twice using BG11_0_ medium and resuspended in 20 mL BG11_0._ The washed cells were split into two flasks, each containing 10 mL cell suspension. In parallel, a single colony of Het4 was picked from a R2A (CL01.1, Carl Roth, Karlsruhe, Germany) plate, transferred to 4 mL LB medium (X968.4, Carl Roth, Karlsruhe, Germany) and incubated at 28 °C, 210 rpm shaking for 14 h. The cells were harvested, washed twice with BG11_0_ medium, resuspended in 2 mL BG11_0_ medium (OD600 = 2.3) and added to one of the PCC 73102 cultures.

Both cultures were then kept under continuous illumination of 40 µE without shaking for 7 days at 23 °C. Samples for immunofluorescence microscopy were taken on day 1, day 4 and day 7 by aseptically removing 1 mL cell suspension and replenishing to the original volume with fresh medium. Cells were fixed as described below and stored at −20 °C in PBS (140 mM NaCl, 2.7 mM KCl, 8.0 mM Na_2_HPO_4_, 1.8 mM KH_2_PO_4_, pH = 7.5) with 10% w/v glycerol.

### DNA sequencing

For Sanger sequencing of the heterotrophic bacterial isolates and for Illumina MiSeq 16S rRNA gene amplicon sequencing, the total DNA from KVJ3 and KVJ2 cultures was isolated using a DNA Isolation Kit for Cells and Tissues (Sigma-Aldrich, Taufkirchen, Germany). Universal primers 27F and 1492R were used in the amplification and Sanger sequencing of single bacterial isolates at LGC Genomics GmbH, Berlin, Germany. V3 and V4 regions of the 16S rRNA gene were PCR amplified and 16S rRNA gene amplicons were sequenced by Illumina MiSeq platform at the Institute of Biology, University of Helsinki, Finland. High-quality reads between 10 and 471 bp with unambiguities were analyzed using Mothur v1.39.5^51^ and aligned against the Silva database (Release 138)^52^ with kmer size of 8. Maximum-likelihood phylogenetic tree with bootstrapping of 100 iterations was constructed using the MEGA X software^53^.

For the whole genome PacBio and Illumina hybrid sequencing, high-molecular weight DNA was isolated from KVJ3 and *Agrobacterium tumefaciens* Het4 using phenol-chloroform extraction. Cells were broken using lysozyme in buffer containing 50 mM Tris-HCl, 100 mM EDTA, 0.1 M NaCl (pH = 7) at 37 °C for one hour. Proteinase K and RNAase were added to the cells and incubation was continued at 56 °C for 2 h. DNA was extracted and purified with phenol and chloroform and precipitated with 70% ethanol in the presence of 0.1 M of NaCl. DNA was dissolved in TE buffer and sequenced at the Institute of Biology, University of Helsinki, Finland. PacBio RSII reads were assembled using HGAP3 implemented in SMRT portal with a default genome size of 6 Mbp and circulated using Gap4. Illumina MiSeq reads were filtered by cutadapt v 1.14, m = 100, q = 25^54^ and genome was polished using bwa v0.7.12-r1039^55^ and Pilon v1.16^56^ with default parameters. Newly sequenced genomes were annotated using NCBI prokaryotic genome annotation pipeline (PGAP)^57^.

High-quality reads were mapped using BWA-MEM (v0.7.17.1)^55^. Reads < 20 bp, secondary aligned reads and PCR duplicates were removed using BAM filter (v0.5.9)^58^ and features were called using featureCounts (v1.6.4)^59^ with paired-end mode and exclusion of chimeric structures. Reads mapped to Het4 genome were removed from the differential gene expression analysis. DESeq2 (v1.28.1)^60^ with rlog transformation was used to call the differentially expressed (DE) genes.

### Preparation of endoproteome and exoproteome fractions

To obtain the exoproteome fraction, cultures were centrifuged for 10 min at 5000 × *g*. The supernatant was removed, centrifuged again for 10 min at 10,000 × *g* and transferred to a fresh microcentrifuge tube. Pre-cultures for the preparation of endoproteomics co-cultivation plates were grown and washed as described above. Subsequently, three 50 µL droplets of either PCC 73102 were spotted on freshly prepared BG110 plates. For the co-cultivation plates, 50 µL of Het4 were gently dropped onto the PCC 73102 spots. Plates were incubated for 16 d under the same conditions as described above. Endoproteome fractions were obtained by scraping culture spots from their respective agar plates and then resuspending the biomass in 300 µL of lysis buffer (25 mM Tris-HCl, 5% (w/v) glycerol, 1% (v/v) Triton-X 100, 1% (w/v) sodium deoxycholate, 0.1% (w/v) sodium dodecyl sulfate (SDS) and 1 mM EDTA). Zirconium oxide beads (diameter 0.15 mm) were added to the cell suspension and cells were broken in a Bullet Blender Storm 24 (Next Advance) with three cycles of 5 min. Cell lysates were centrifuged at 10,000 × *g* for 10 min and the resulting supernatant transferred for further analysis. The protein content of all samples was determined with a PierceTM BCA assay kit (Thermo Fisher Scientific).

### Proteomic analysis

Sample preparation for proteomic analysis was done according to the EXCRETE workflow^37^. Briefly, the equivalent of 10 µg of protein was harvested from the supernatant and transferred to 2 mL microcentrifuge tubes. NaCl and SDS were added to a final concentration of 10 mM and 1% (w/v) and samples were reduced with 5 mM TCEP and alkylated with 5.5 mM CAA. Protein aggregation was induced by adding LC-MS grade ethanol to a final concentration of 50% (v/v) followed by SiMAG-Carboxyl magnetic particles (product no. 1201, Chemicell) to a final concentration of 0.5 µg µL^−^^1^. Samples were incubated for 10 min with shaking at 1000 rpm and, subsequently, magnetic particles were separated on a magnetic rack and washed, on-magnet, 3 times with 80% (v/v) ethanol. Protein digestion was done overnight at 37 °C on-bead in 100 µL of 25 mM ammonium bicarbonate containing 0.5 µg of MS grade Trypsin/LysC (Promega) (enzyme/protein ratio of 1:20 (w/w)). Following protein digestion, magnetic particles were separated for 60 s and supernatants were recovered. Peptide purification and desalting, LC-MS analysis and raw data processing were done as previously described (Russo et al. ^37^). For protein identification a protein group was considered identified when it was present in at least 70% of the replicates with a minimum of three replicates. Missing values were imputed using the k-nearest neighbors’ algorithm. Proteins were annotated using EggNOG v5.0 (Huerta-Cepas et al.^62^), PsortB v3.0 (Yu et al.^63^), SignalP 6.0 (Teufel et al.^64^) and UniProtKB (The UniProt Consortium^65^). Data analysis and visualization were performed using custom scripts in R (4.3.0) with packages ggrepel (0.9.5), ggplot2 (3.5.1), viridis (0.6.5), readxl (1.4.3) and gplots (3.1.3.1). Differential protein analysis was done using a Student’s two-sample unpaired t-test with permutation-based multiple test correction with a cutoff criterion of fold change = 2 and adjusted *p*-value < 0.05.

### Immunofluorescence microscopy

Cells were harvested by centrifugation and washed twice in freshly prepared PBS. Washed cells were resuspended in 4% w/v paraformaldehyde in PBS (J61899.AK, Thermo Fisher, Hennigsdorf, Germany), incubated for 10 min at RT, washed two more times with PBS, resuspended in ultra-pure water and subsequently spread on 2H coverslips (01-0012/2, Langenbrinck, Emmendingen, Germany) to air-dry until sufficient attachment was achieved without completely desiccating the cells. All following steps were carried out in a humidifier.

The specimens were washed once with PBS for 5 min, then permeabilized using 2 mg/mL of lysozyme (0663-10 G, VWR, Darmstadt, Germany) in PBS with 0.3% w/v Triton-X 100 (PBS-TX) for 30 min at RT, washed twice in PBS-TX for 3 min, blocked using 1% w/v Polyvinylpyrrolidone K30 (4607.1, Carl Roth, Karlsruhe, Germany) in PBS with 0.3% w/v Tween-20 (PBS-T) for 1 h at 4 °C and washed twice in PBS-T for 3 min.

For antibody labeling, the specimen was incubated for 1 h at RT with primary antibodies (rabbit anti-rbcL large subunit, form I polyclonal antibody, AS03 037 A, Agrisera, Vännas, Sweden; for negative controls: rabbit anti-GFP N-terminal polyclonal antibody, Sigma-Aldrich, G1544) diluted to 4 µg/ml in PBS-T, and washed twice in PBS-T for 3 min.

Secondary antibodies (goat-anti-rabbit IgG Alexa Fluor 488, A-11008, Thermo Fisher, Hennigsdorf, Germany) were diluted 1:200 in PBS-T and applied by incubation for 1 h at 4 °C in the dark. After two final washes in PBS-T, specimens were air-dried and mounted on slides using ProLong Glass (P36980, Thermo Fisher, Hennigsdorf, Germany). Slides were imaged using a Zeiss LSM780 (Carl Zeiss Microscopy, Jena, Germany) laser scanning confocal microscope equipped with a Plan-Apochromat 63×/1.40 oil immersion lens. Alexa Fluor 488 was excited at 488 nm and detected at 493–550 nm, whereas autofluorescence was excited at 633 nm, and detected between 647 and 687 nm.

### Electron microscopy

Cells from 2 mL of PCC 73102 culture were collected, washed, and fixed by 2.5% glutaraldehyde, 2.0% formaldehyde in 0.1 M Na–cacodylate buffer, pH 7.4 similarly as described earlier^42^. Subsequently, samples were overlaid by a thin layer of 1% low-melting agarose, dehydrated in a graded EtOH series and acetone and embedded in low viscosity resin (Agar Scientific, Stansted, Essex, UK). Ultrathin sections stained with uranyl acetate and lead citrate were examined in a Talos F200C transmission electron microscope (Thermo Fisher Scientific, Waltham, MA, USA), operated at 200 kV.

### Immunogold electron microscopy

Cells were carefully removed from agar plates, resuspended in 1 mL PBS, and treated as described previously in the “Immunofluorescence microscopy” section until secondary antibody hybridization. After washing in PBS, specimens were incubated for 60 min with 10-nm-gold-conjugated goat-anti-rabbit IgG (Sigma-Aldrich, product number G7402), diluted 1:25 in PBS, and washed in PBS for 2 × 5 min. After a further 5-min-washing step in PBS supplemented with 0.5 M NaCl, specimens were incubated for 5 min in 0.1 M Na-phosphate buffer (pH 7.3), post-fixed with phosphate-buffered 3% glutaraldehyde for 30 min, washed in phosphate buffer for 5 min, and dehydrated in a graded EtOH series and acetone. Coverslips were then embedded in Spurr low viscosity resin (Science Services, Munich, Germany) as described in detail previously^61^. After polymerization, coverslips were removed from the polymerized resin by several cycles of cooling (liquid N_2_) and re-warming to room temperature. Areas with bacteria were selected by phase contrast microscopy, mounted^61^, sectioned on an ultramicrotome at a thickness of 90 nm, stained with uranyl acetate for 5 min, and analyzed in a Talos F200C.

## Supplementary information

Supplemental Material

Dataset S1

Dataset S2

## Data Availability

The authors confirm that sequencing data supporting the findings of the study are publicly available in the NCBI database [SRA accession numbers SRR24556967 and SRR24556966 and project numbers PJNA599284 and PRJNA599316]. The mass spectrometry proteomics data have been deposited to the ProteomeXchange Consortium via the PRIDE partner repository with the dataset identifier PXD052511.
