# Supplementary material for: Competition and interdependence define interactions of Nostoc sp. and Agrobacterium sp. under inorganic carbon limitation
Source: NPJ Biofilms Microbiomes. 2025 Mar 8;11:42. doi: 10.1038/s41522-025-00675-0 (PMC11890772; doi:10.1038/s41522-025-00675-0)
Supplement: Supplementary file 1 — Supplemental Material [file 41522_2025_675_MOESM1_ESM.pdf]

Supporting information

Competition and interdependence define interactions of *Nostoc* sp. and *Agrobacterium* sp. under inorganic carbon limitation

Jonna E. Teikari, David A. Russo, Markus Heuser, Otto Baumann, Julie A. Z. Zedler, Anton Liaimer, Elke Dittmann

Table of Contents:

Supplementary Tables:

Supplementary Table 1 Genome analysis of *N. punctiforme* PCC 73102 and *Nostoc* sp. KVJ2 and KVJ3 to identify genes related to the cyanobacterial carbon concentrating mechanism (CCM).
Supplementary Table 2 Results of the pairwise t-Tests conducted to determine significant differences
in Surface Greenness (Figure S3).

Supplementary Figures:

Supplementary Figure 1 Phylogenetic comparison of SbtA-orthologues and SbtA-like proteins from *Nostoc* and selected other cyanobacterial strains.
Supplementary Figure 2 Phylogenetic tree based on 16S rRNA V3-V4 regions.
Supplementary Figure 3 Heterotrophic bacterial isolates promote growth of *N. punctiforme* PCC 73102.
Supplementary Figure 4 Digital image analysis of growth promotion assays.
Supplementary Figure 5 Amino acid identity (AAI) of *A. tumefaciens* Het4 illustrated by AAI profiler.
Supplementary Figure 6 Volcano plot illustrating differential expression of all proteins identified in the *N. punctiforme* PCC 73102 endoproteome in monoculture and co-culture with *A. tumefaciens* Het4.
Supplementary Figure 7 Heatmap illustrating the differential expression of *N. punctiforme* PCC 73102 proteins related to mobility and chemotaxis between a monoculture and co-culture with *Agrobacterium tumefaciens* Het4.
Supplementary Figure 8 Immunofluorescence labelling of carboxysomal shell protein CcmK in *N. punctiforme* PCC 73102.
Supplementary Figure 9 Negative control for immunofluorescence staining.
Supplementary Figure 10 Intensity of proteins identified in the *N. punctiforme* PCC 73102 exoproteome ordered by rank.
Supplementary Figure 11 Immunofluorescence detection of RbcL under nitrogen replete conditions in BG11 medium.
Supplementary Figure 12 Immunogold TEM. Co-culture of *N. punctiforme* PCC 73102 and *A. tumefaciens* Het4 was incubated with anti-RbcL and, for control, anti-GFP.

Supplementary Tables

Supplementary Table 1
Genes related to cyanobacterial carbon concentrating mechanism (CCM). The following genome and metagenome sequence data were used for the analysis: PCC 73102 (GCA_000020025.1), KVJ2 (SAMN07173937) and KVJ3 (PRJNA599284).

Gene product	PCC 73102	KVJ2	KVJ3	Reference
BicA	+	+	+	(Sandrini *et al*, 2014)
SbtA	-	-	-	(Sandrini *et al.*, 2014)
SbtB	-	-	-	(Sandrini *et al.*, 2014)
CcmR2	+	+	+	(Sandrini *et al.*, 2014)
SbtA-like	+	+	+	(Shibata et al., 2002)
nhaS3	+	+	+	(Sandrini *et al.*, 2014)
CmpA	+	+	+	(Sandrini *et al.*, 2014)
CmpB	+	+	+	(Sandrini *et al.*, 2014)
CmpC	+	+	+	(Sandrini *et al.*, 2014)
CmpD	+	+	+	(Sandrini *et al.*, 2014)
EcaA	-	-	-	(Sandrini *et al.*, 2014)
EcaB	-	-	-	(Sandrini *et al.*, 2014)
CcaA (icfA)	+	+	+	(So & Espie, 2005)
CcmM	+	+	+	(So & Espie, 2005)
NdhD3	+	+	+	(Shibata *et al*, 2001)
NdhF3	+	+	+	(Shibata *et al.*, 2001)
CupA	+	+	+	(Shibata *et al.*, 2001)
NdhD4	+	+	+	(Shibata *et al.*, 2001)
NdhF4	+	+	+	(Shibata *et al.*, 2001)
CupB	+	+	+	(Shibata *et al.*, 2001)

Supplementary Table 2 Results of the pairwise t-Tests conducted to determine significant differences in Surface Greenness (**Supplementary** Figure 4).

Day	Condition	Δ(Mean Greenness)	Mean Greenness Nostoc	Mean Grenness Nostoc + Het4	Test statistic	p Value	Degrees of Freedom
0	High	2.52456	11.56156	9.03700	0.54690	5.93E-01	13.71682
	Low	-12.18278	10.44856	22.63133	-1.50679	1.65E-01	9.20178
	Medium	8.88878	19.89278	11.00400	1.12142	2.86E-01	11.23541
1	High	5.18933	11.54200	6.35267	1.87748	8.37E-02	12.65808
	Low	-0.76300	1.71644	2.47944	-0.98809	3.39E-01	15.25803
	Medium	-21.49478	1.68133	23.17611	-10.42848	4.10E-06	8.45300
3	High	50.90411	75.13944	24.23533	7.74294	5.65E-06	11.85538
	Low	24.70300	32.14044	7.43744	9.15936	9.76E-07	11.90168
	Medium	20.34489	25.14811	4.80322	11.35697	9.29E-08	11.94898
5	High	76.04422	124.90444	48.86022	10.63478	1.01E-07	12.80782
	Low	38.88156	43.91700	5.03544	10.84684	4.69E-07	10.55758
	Medium	-18.67167	43.17433	61.84600	-4.25610	6.04E-04	15.99711
7	High	47.31600	157.98589	110.66989	4.39186	4.90E-04	15.47384
	Low	31.18111	48.27478	17.09367	6.22785	7.73E-05	10.55796
	Medium	-47.34278	57.12156	104.46433	-7.04424	3.04E-06	15.73557
9	High	24.33922	179.83044	155.49122	3.50506	2.96E-03	15.89005
	Low	-18.46256	37.53856	56.00111	-3.35114	5.79E-03	11.96211
	Medium	-86.88833	60.23356	147.12189	-12.64963	3.24E-08	11.79074

Supplementary Figures

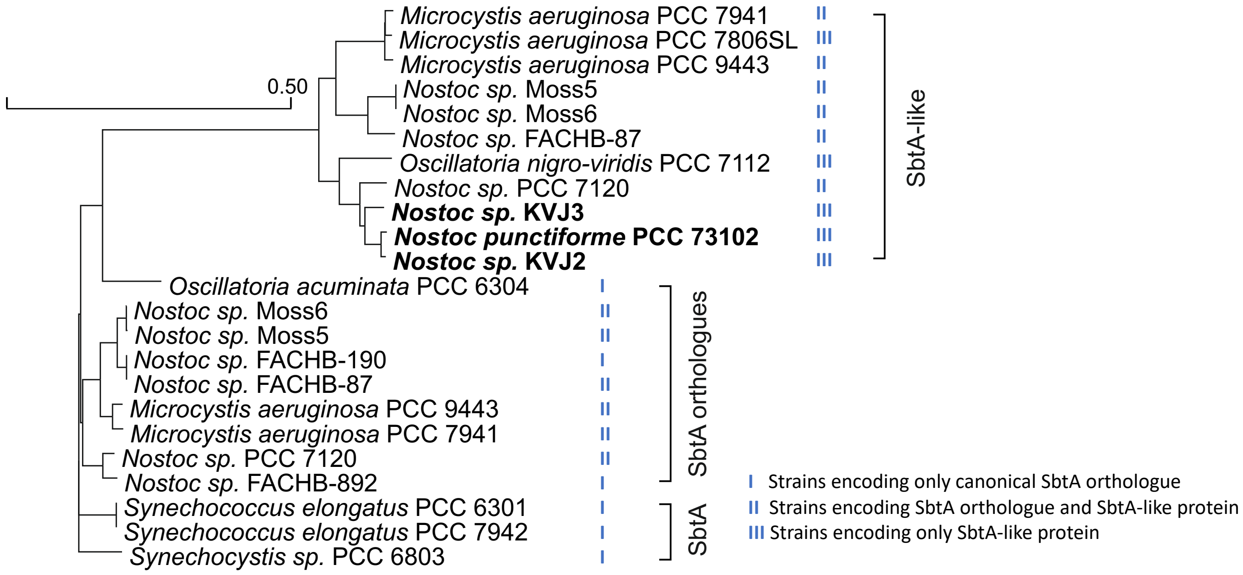

Supplementary Figure 1 Phylogenetic comparison of SbtA orthologues and SbtA-like proteins from *Nostoc* and selected other cyanobacterial strains. *N. punctiforme* PCC 73102, *Nostoc* sp. KVJ2 and *Nostoc* sp. KVJ3 encode an SbtA-like protein, but not the well characterized SbtA orthologue which is highly conserved in the majority of cyanobacteria (Shibata *et al.* 2001). Most cyanobacterial strains encode either solely the canonical SbtA protein (indicated by roman number I) or both the canonical SbtA orthologue and the SbtA-like protein (indicated by roman number II). A few cyanobacterial strains again only encode the SbtA-like protein such as *Nostoc punctiforme* PCC 73102, KVJ2 and KVJ3 which are highlighted in bold and *M.aeruginosa* PCC 7806SL (indicated by roman number III). *Microcystis* strains lacking the canonical SbtA and only encoding the SbtA-like protein like *M. aeruginosa* PCC 7806SL show a weak carbon-concentrating mechanism compared to strains carrying both copies (Sandrini *et al.* 2014).

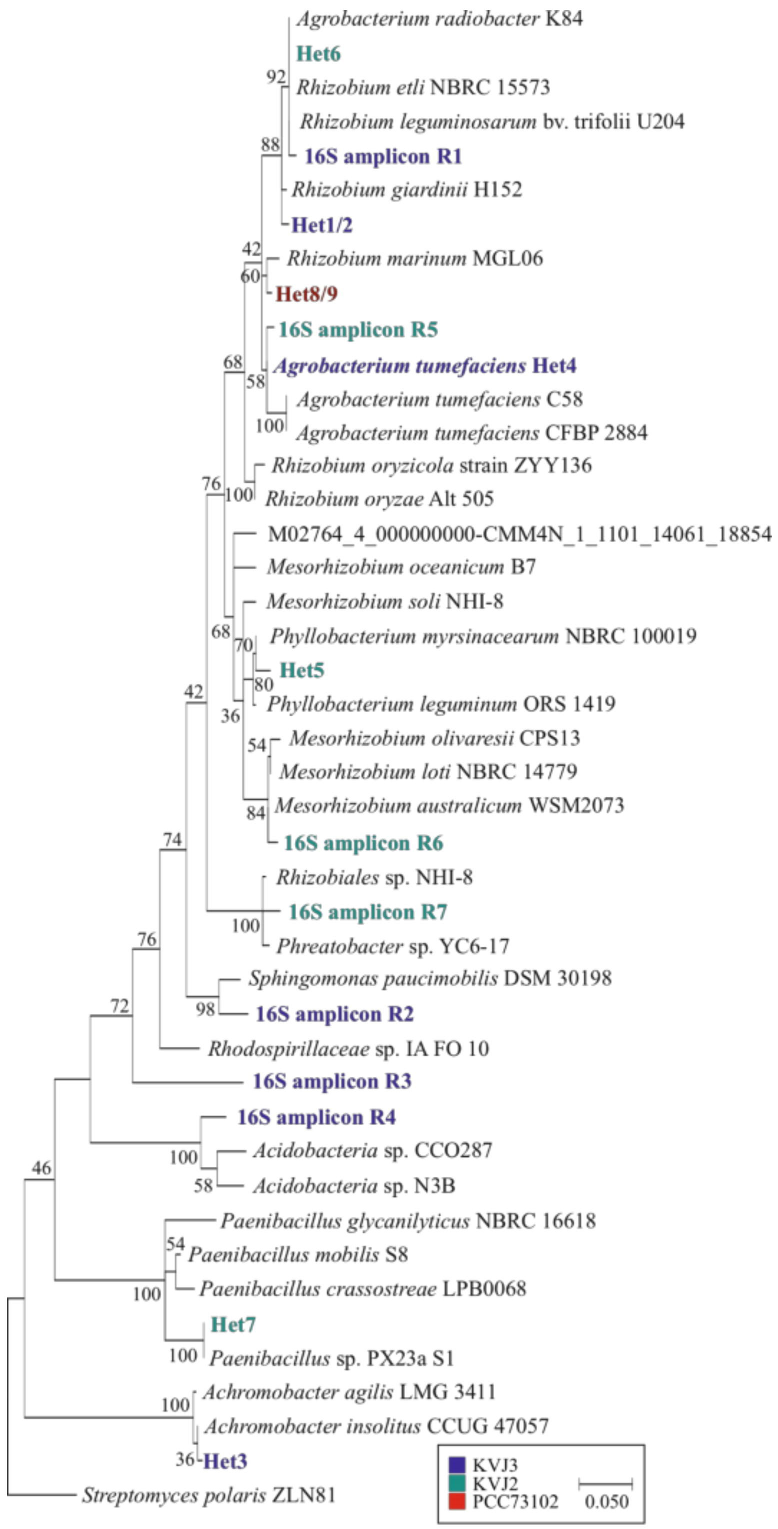

Supplementary Figure 2 Phylogenetic tree based on 16S rRNA V3-V4 regions. Most of the isolated heterotrophic bacteria belonged to the *Rhizobium*/*Agrobacterium* group. Het8/9 represent strains isolated from incidentally contaminated *N. punctiforme* PCC 73102. In addition, the four most abundant amplicons from 16S rRNA amplicon sequencing from the *Nostoc* sp. KVJ3 and three from the *Nostoc* sp. KVJ2 microbiome were included in the tree.

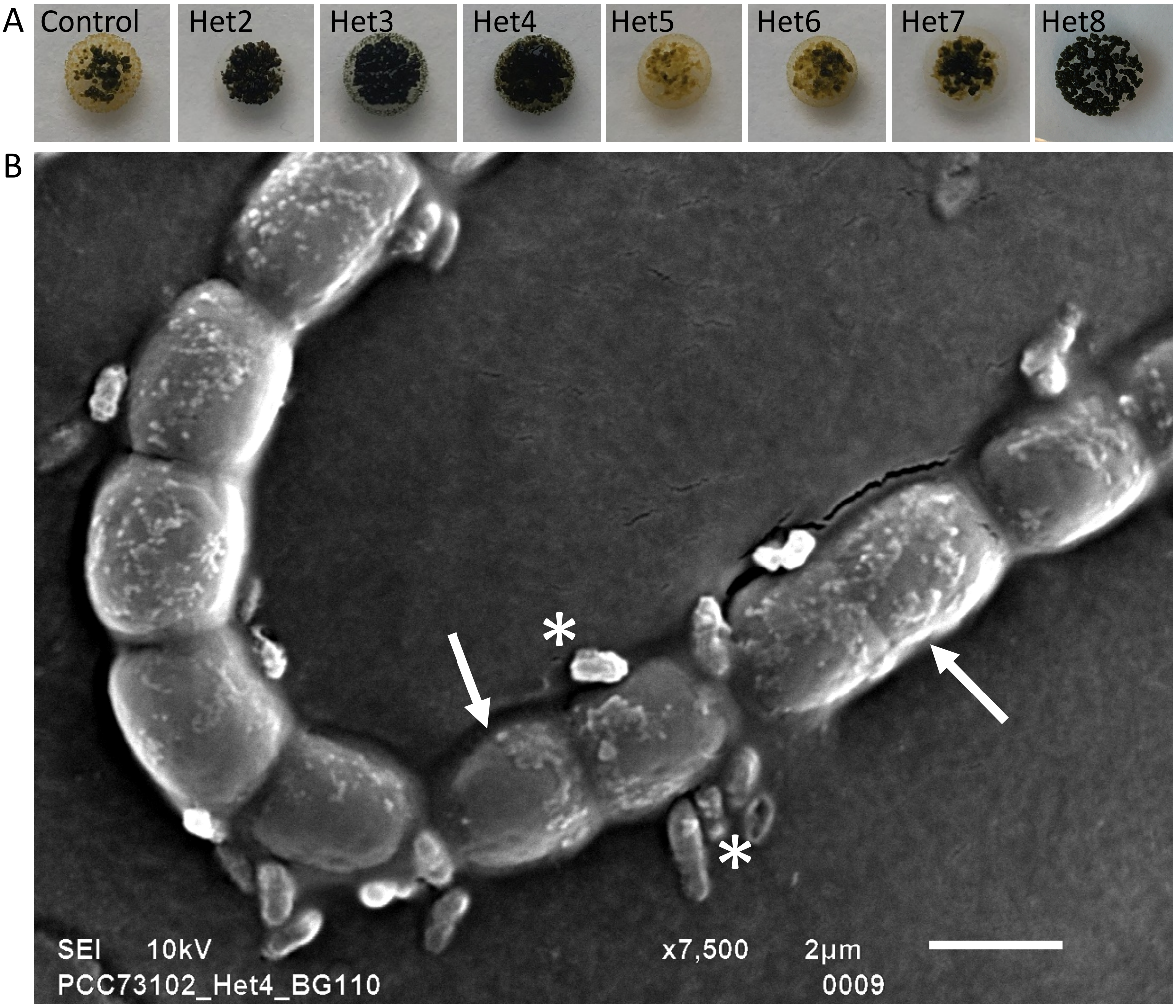

Supplementary Figure 3 Heterotrophic bacterial isolates promote growth of *N. punctiforme* PCC 73102. (A) Several isolated heterotrophic bacteria enhanced the growth of *N. punctiforme* PCC 73102 on $BG11_0$ agar plates. Particularly Het 3, 4 and 8 had a strong growth-promoting effect on the *N. punctiforme* PCC 73102. (B) Scanning electron microscopy showed an intimate interaction with *A. tumefaciens* Het4 (asterisk) and remarkable number of heterotrophic bacteria on the cell wall of cyanobacteria (arrow).

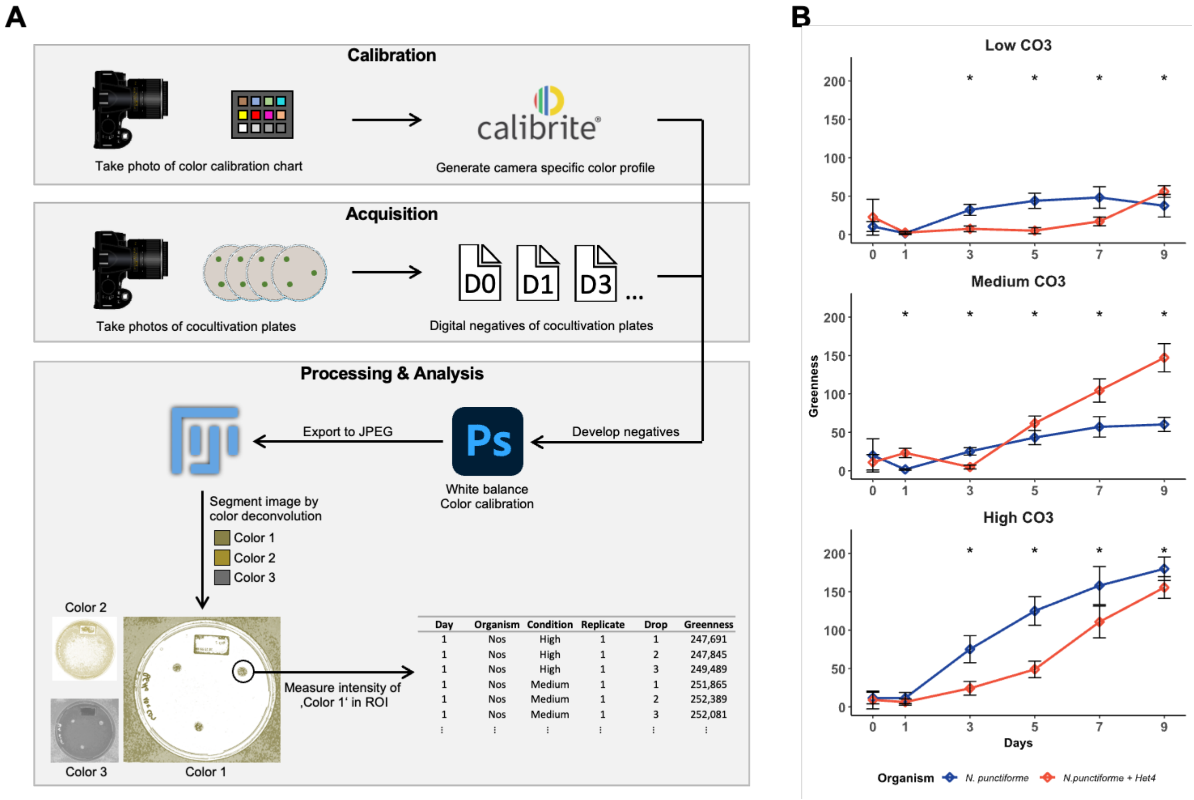

Supplementary Figure 4 Digital image analysis of growth promotion assays A) Graphical overview of the measuring method to determine Surface Greenness. In short, first a color profile for the particular camera used to take the pictures is generated using a commercially available color target and the accompanying software. Then, this color profile is used to color correct the RAW photos taken of the cultivation plates while also performing white-balancing. From a representative plate picture the values for color deconvolution and the measuring area are recorded using their respective UIs in Fiji. These values are subsequently used on every plate picture to generate the table of measurements. Data visualization is done in RStudio. Image sources: The Calibrite Logo is a registered trademark of Calibrite LLC, Wilmington, USA. petri-dish-top-gray icon by Servier https://smart.servier.com/ (CC-BY 4.0). (B) Surface Greenness measurements of axenic *N. punctiforme* PCC 73102 and *N. punctiforme* PCC 73102 + *A. tumefaciens* Het4 co-cultivation plates und various carbonate conditions. The droplet plates were prepared and incubated as previously described. Measured Surface Greenness in time points marked with an asterisk is statistically significantly different, as determined by *Welchs Two Sample t-test* using a $p < 0.05$ cutoff. T-test results are shown in Table S2.

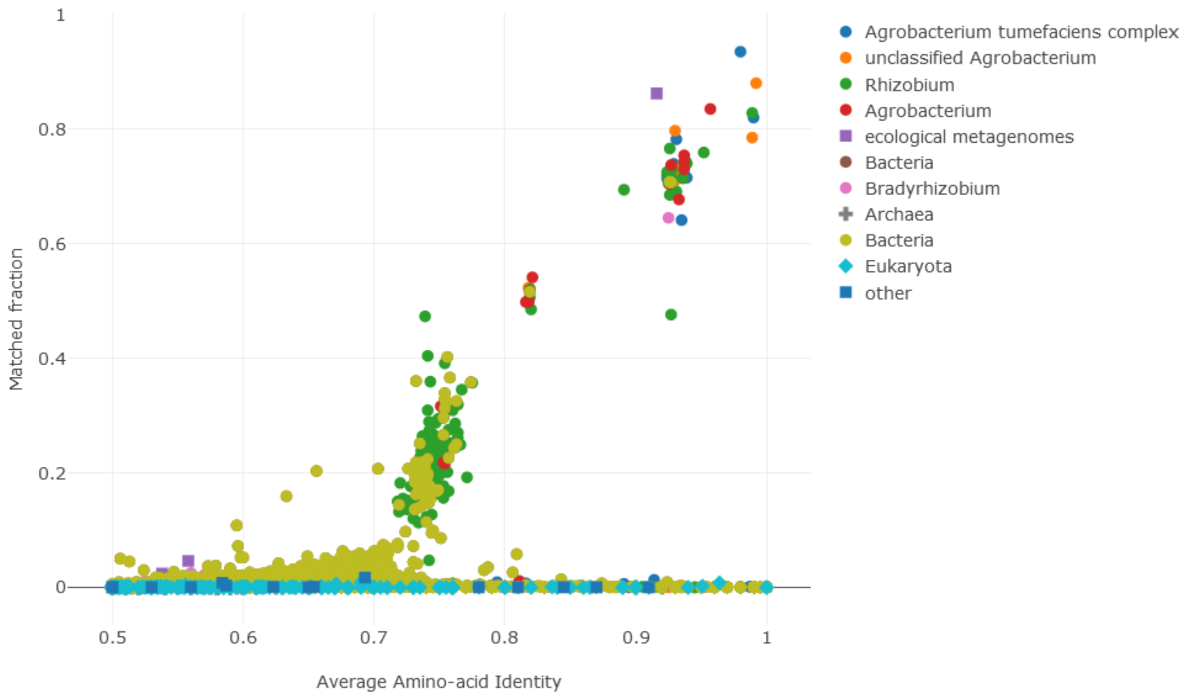

Supplementary Figure 5 Amino acid identity (AAI) of *A. tumefaciens* Het4 illustrated by AAI profiler (Medlar *et al*, 2018). AAI is plotted on the horizontal axis and the vertical axis represents the matched fraction of query proteins to the database. AAI values above 95% correspond to the same species.

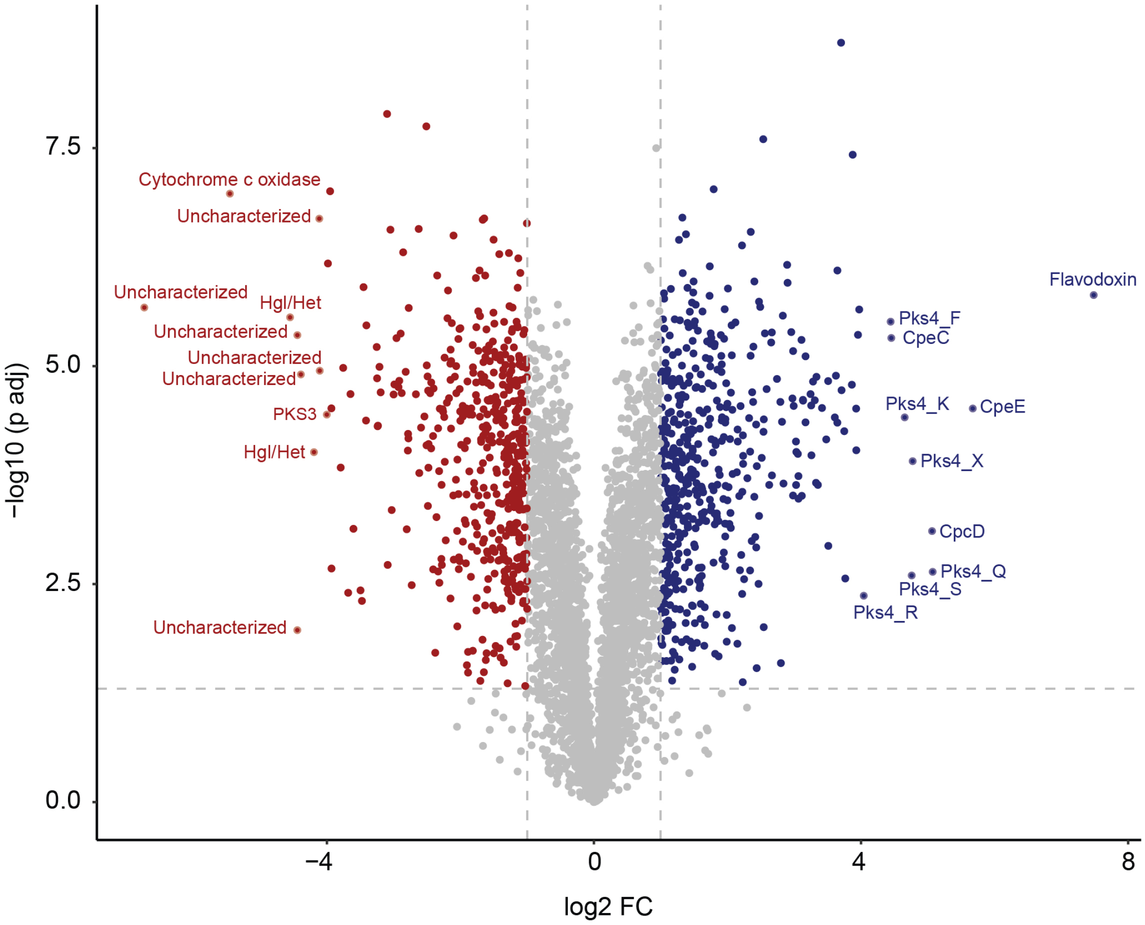

Supplementary Figure 6 Volcano plot illustrating differential expression of all proteins identified in the *N. punctiforme* PCC 73102 endoproteome in monoculture and co-culture with *A. tumefaciens* Het4. Each dot on the plot represents an individual protein and labels indicate protein name, when available. A Student's two-sample unpaired t-test with permutation-based multiple test correction was used for differential protein analysis. The fold-change and significance thresholds are indicated by vertical and horizontal dashed lines, respectively. Color coding indicates significant changes (red for downregulated, blue for upregulated, and grey for non-significant changes).

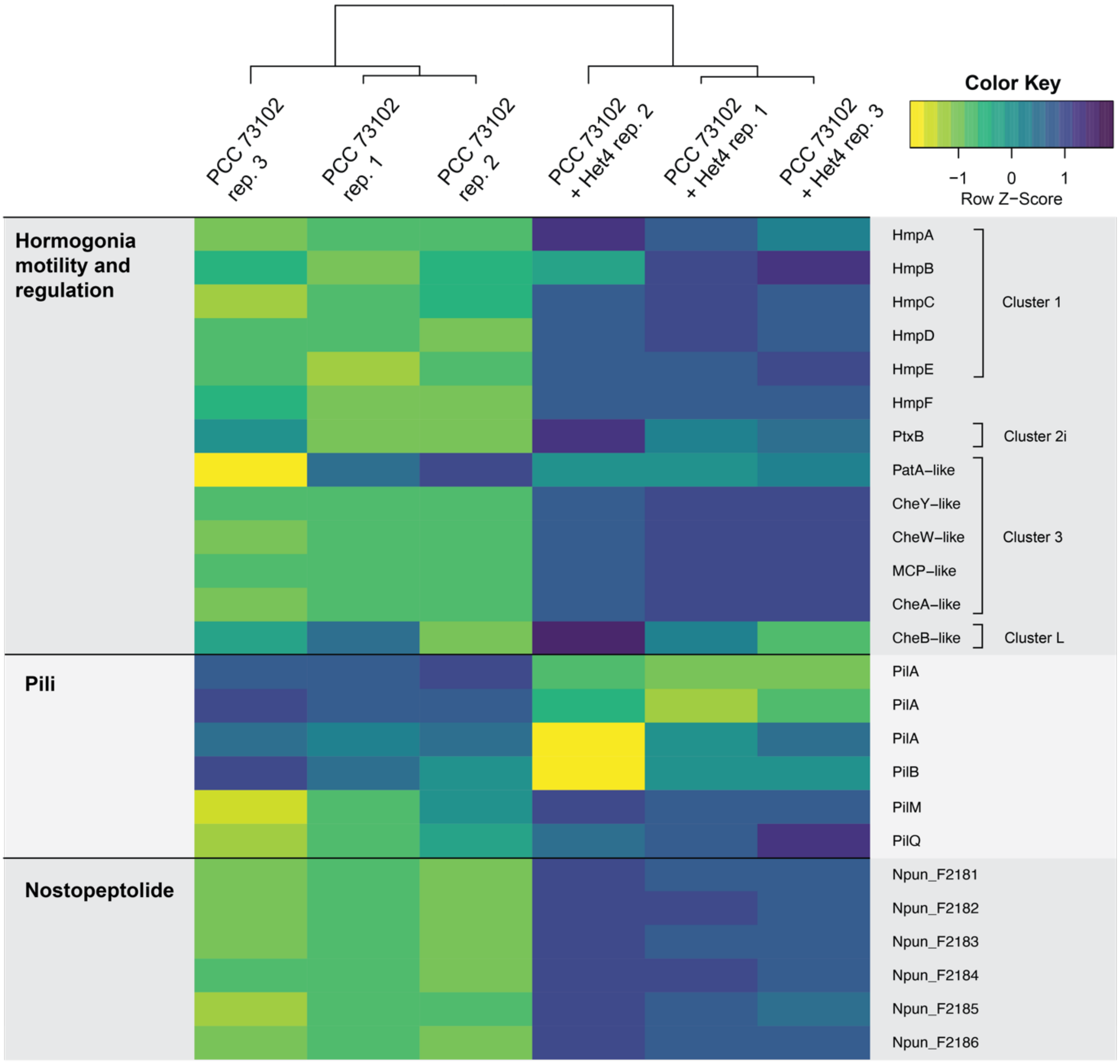

Supplementary Figure 7 Heatmap illustrating the differential expression of *N. punctiforme* PCC 73102 proteins related to mobility and chemotaxis between a monoculture and co-culture with *Agrobacterium tumefaciens* Het4. Rows represent individual proteins, while columns represent biological replicates of each condition. Proteins are labelled with protein names (right) and grouped by predicted cellular function (left). Cluster names of motility related protein refer to the nomenclature established in (Campbell *et al*, 2015). Log2 protein expression levels were transformed with a z-score normalization and represented with a color scale that varies from yellow (most downregulated) to blue (most upregulated) relative to the mean expression across all samples in each row. Hierarchical clustering was performed on samples and dendrograms indicate the similarity between them.

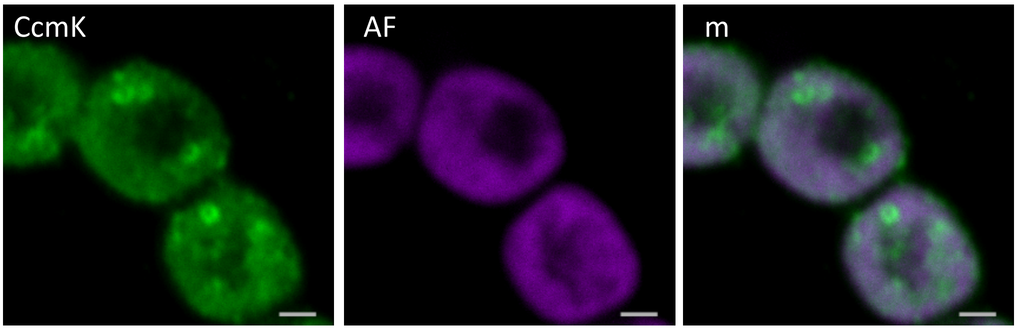

Supplementary Figure 8 Immunofluorescence labelling of carboxysomal shell protein CcmK in *N. punctiforme* PCC 73102. Immunofluorescence labeling for CcmK and RbcS in *N. punctiforme* PCC 73102. Carboxysome shell protein (CcmK2) was used to serve as a marker protein to map the placement of the carboxysomes in the cells. Typical carboxysomal ring-like structure also confirmed the integrity of the filaments in the specimen. AF = autofluorescence, m = merged images. Scale bar 1 µm.

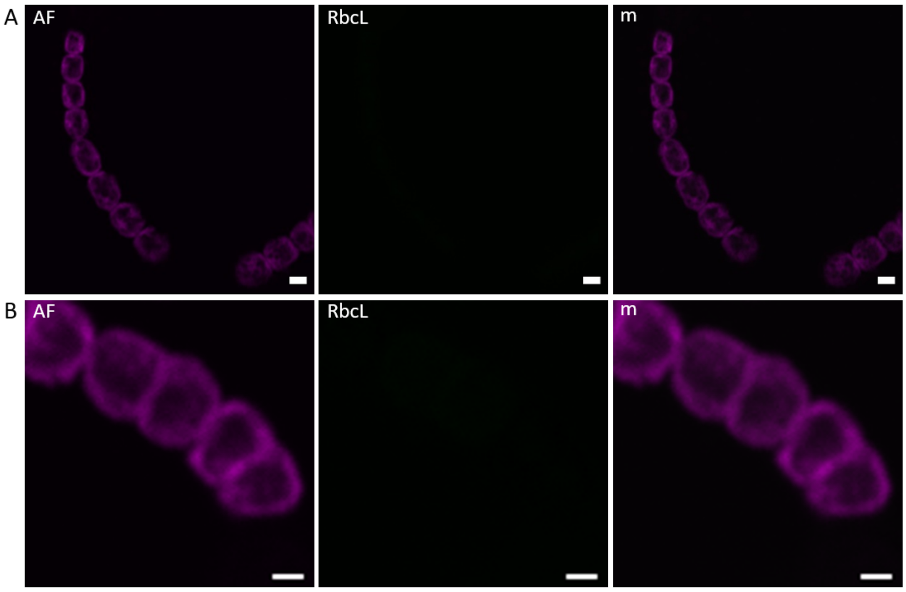

Supplementary Figure 9 Negative controls for IFM. Images were acquired by using the same parameters as in Fig. 3 and Fig. S6. Images show no binding of the secondary antibody in axenic *N. punctiforme* PCC 73102 grown on $BG11_0$. AF = phycobilisome autofluorescence, m = merged. (A) Scale bar 2 µm (B) Scale bar 1 µm.

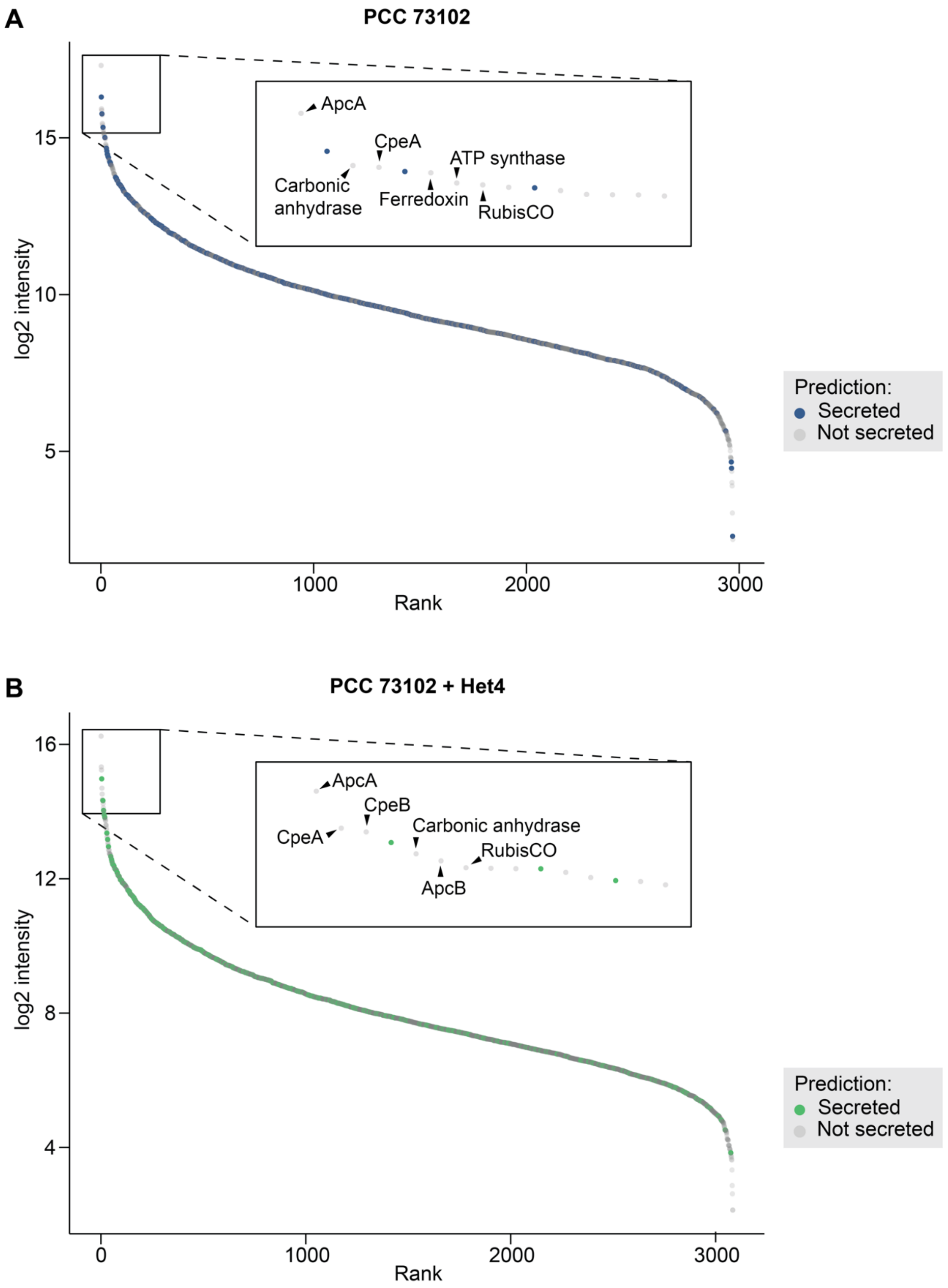

Supplementary Figure 10 Intensity of proteins identified in the *N. punctiforme* PCC 73102 exoproteome ordered by rank. The color indicates whether a protein is predicted to be actively secreted (secreted, blue (A)/ green dots (B)) or present via alternative routes (not secreted, grey dots). Panel A represents the exoproteome of *N. punctiforme* PCC 73102 in monoculture. Panel B represents the exoproteome of *N. punctiforme* PCC 73102 in co-culture with *A. tumefaciens* Het4. Secretion prediction was done according to (Russo *et al*, 2024). Dots represent means of biological replicates. Insets show the top 15 most abundant proteins

identified in the exoproteome with labels indicating the 6 most abundant proteins predicted as not secreted.

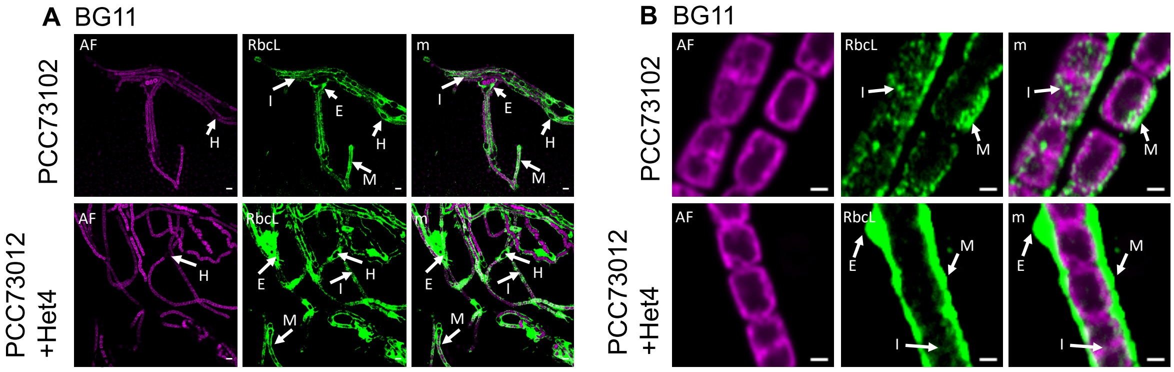

Supplementary Figure 11 Immunofluorescence detection of RbcL under nitrogen replete conditions in BG11 medium (A) Overview micrographs illustrating the phenotypic heterogeneity among filaments under nitrogen replete conditions. (B) Selected detail images showing subcellular localization of RbcL in mono-and co-cultures.

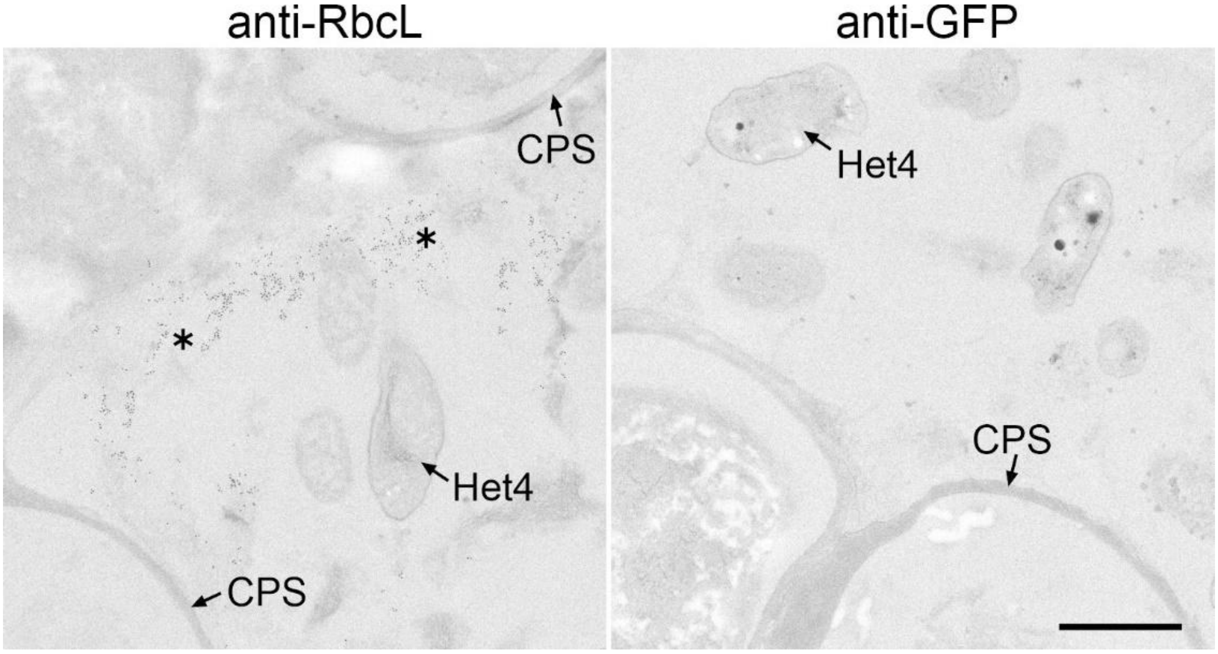

Supplementary Figure 12 Immunogold TEM. Co-culture of *N. punctiforme* PCC730102 and *A. tumefaciens* Het4 was incubated with anti-RbcL and, for control, anti-GFP. Note the numerous immunogold particles (asterisks) at the periphery of the polysaccharide capsid (CPS) in the specimen incubated with anti-RbcL. Lack of immunogold in the control specimen demonstrates specificity of anti-RbcL labeling. Bar, 1 µm

References

Campbell EL, Hagen KD, Chen R, Risser DD, Ferreira DP, Meeks JC (2015) Genetic analysis reveals the identity of the photoreceptor for phototaxis in hormogonium filaments of *Nostoc punctiforme*. *J Bacteriol* 197: 782-791

Medlar AJ, Toronen P, Holm L (2018) AAI-profiler: fast proteome-wide exploratory analysis reveals taxonomic identity, misclassification and contamination. *Nucleic Acids Res* 46: W479-W485

Russo DA, Oliinyk D, Pohnert G, Meyer F, Zedler JAZ (2024) EXCRETE enables deep proteeomics of the microbial extracellular environment. *Commun. Biol.* 7: 1189.

Sandrini G, Matthijs HCP, Verspagen JMH, Muyzer G, Huisman J (2014) Genetic diversity of inorganic carbon uptake systems causes variation in CO2 response of the cyanobacterium *Microcystis*. *ISME J* 8: 589-600

Shibata M, Ohkawa H, Kaneko T, Fukuzawa H, Tabata S, Kaplan A, Ogawa T (2001) Distinct constitutive and low-CO2-induced CO2 uptake systems in cyanobacteria: genes involved and their phylogenetic relationship with homologous genes in other organisms. *Proc Natl Acad Sci U S A* 98: 11789-11794

Shibata, M. *et al.* (2002) Genes essential to sodium-dependent bicarbonate transport in cyanobacteria: function and phylogenetic analysis. *J. Biol. Chem.* **277**, 18658-18664, doi:10.1074/jbc.M112468200.

So AKC, Espie GS (2005) Cyanobacterial carbonic anhydrases. *Can J Bot* 83: 721-734
